# Polar Ocean Observations: A Critical Gap in the Observing System and Its Effect on Environmental Predictions From Hours to a Season

**DOI:** 10.3389/fmars.2019.00429

**Published:** 2019-08-06

**Authors:** Gregory C. Smith, Richard Allard, Marcel Babin, Laurent Bertino, Matthieu Chevallier, Gary Corlett, Julia Crout, Fraser Davidson, Bruno Delille, Sarah T. Gille, David Hebert, Patrick Hyder, Janet Intrieri, José Lagunas, Gilles Larnicol, Thomas Kaminski, Belinda Kater, Frank Kauker, Claudie Marec, Matthew Mazloff, E. Joseph Metzger, Calvin Mordy, Anne O’Carroll, Steffen M. Olsen, Michael Phelps, Pamela Posey, Pierre Prandi, Eric Rehm, Phillip Reid, Ignatius Rigor, Stein Sandven, Matthew Shupe, Sebastiaan Swart, Ole Martin Smedstad, Amy Solomon, Andrea Storto, Pierre Thibaut, John Toole, Kevin Wood, Jiping Xie, Qinghua Yang, WWRP PPP Steering Group

**Affiliations:** 1 Environmental Numerical Prediction Research Section, Meteorological Research Division, Environment and Climate Change Canada, Dorval, QC, Canada; 2 Stennis Space Center, U.S. Naval Research Laboratory, Bay St. Louis, MS, United States; 3 Takuvik, UMI 3376, Université Laval-CNRS, Quebec City, QC, Canada; 4 Nansen Environmental and Remote Sensing Center, Bergen, Norway; 5 Division of Marine and Oceanography, Météo France, Toulouse, France; 6 CNRM, Météo France, CNRS, Université de Toulouse, Toulouse, France; 7 European Organisation for the Exploitation of Meteorological Satellites, Darmstadt, Germany; 8 Perspecta, Inc., Stennis Space Center, Bay St. Louis, MS, United States; 9 Northwest Atlantic Fisheries Centre, Fisheries and Oceans Canada, St. John’s, NL, Canada; 10 Chemical Oceanography Unit, Université de Liège, Liège, Belgium; 11 Scripps Institution of Oceanography University of California, San Diego, La Jolla, CA, United States; 12 Met Office, Exeter, United Kingdom; 13 Physical Sciences Division, NOAA Earth System Research Laboratory, Boulder, CO, United States; 14 Collecte Localisation Satellites, Toulouse, France; 15 The Inversion Lab, Hamburg, Germany; 16 Arcadis Nederland B.V., Zwolle, Netherlands; 17 Ocean Atmosphere Systems, Hamburg, Germany; 18 Alfred Wegener Institute for Polar and Marine Research, Bremerhaven, Germany; 19 Laboratoire d’Oceanographie Physique et Spatiale, UMR 6523, CNRS – IFREMER – IRD – UBO, Plouzané, France; 20 Joint Institute for the Study of the Atmosphere and Oceans, University of Washington, Seattle, WA, United States; 21 European Organisation for the Exploitation of Meteorological Satellites, Darmstadt, Germany; 22 Danish Meteorological Institute, Copenhagen, Denmark; 23 Bureau of Meteorology Hobart, TAS, Australia; 24 Polar Science Center, University of Washington, Seattle, WA, United States; 25 Cooperative Institute for Research in Environmental Sciences, University of Colorado Boulder, Boulder, CO, United States; 26 Department of Marine Sciences, University of Gothenburg, Gothenburg, Sweden; 27 Department of Oceanography, University of Cape Town, Rondebosch, South Africa; 28 Earth System Research Laboratory, National Oceanic and Atmospheric Administration, Boulder, CO, United States; 29 Centre for Maritime Research and Experimentation, La Spezia, Italy; 30 Woods Hole Oceanographic Institution, Woods Hole, MA, United States; 31 Guangdong Province Key Laboratory for Climate Change and Natural Disaster Studies, School of Atmospheric Sciences, Sun Yat-sen University, Zhuhai, China; 32 World Weather Research Programme (WWRP) Polar Prediction Project (PPP) Steering Group

**Keywords:** polar observations, operational oceanography, ocean data assimilation, ocean modeling, forecasting, sea ice, air-sea-ice fluxes, YOPP

## Abstract

There is a growing need for operational oceanographic predictions in both the Arctic and Antarctic polar regions. In the former, this is driven by a declining ice cover accompanied by an increase in maritime traffic and exploitation of marine resources. Oceanographic predictions in the Antarctic are also important, both to support Antarctic operations and also to help elucidate processes governing sea ice and ice shelf stability. However, a significant gap exists in the ocean observing system in polar regions, compared to most areas of the global ocean, hindering the reliability of ocean and sea ice forecasts. This gap can also be seen from the spread in ocean and sea ice reanalyses for polar regions which provide an estimate of their uncertainty. The reduced reliability of polar predictions may affect the quality of various applications including search and rescue, coupling with numerical weather and seasonal predictions, historical reconstructions (reanalysis), aquaculture and environmental management including environmental emergency response. Here, we outline the status of existing near-real time ocean observational efforts in polar regions, discuss gaps, and explore perspectives for the future. Specific recommendations include a renewed call for open access to data, especially real-time data, as a critical capability for improved sea ice and weather forecasting and other environmental prediction needs. Dedicated efforts are also needed to make use of additional observations made as part of the Year of Polar Prediction (YOPP; 2017–2019) to inform optimal observing system design. To provide a polar extension to the Argo network, it is recommended that a network of ice-borne sea ice and upper-ocean observing buoys be deployed and supported operationally in ice-covered areas together with autonomous profiling floats and gliders (potentially with ice detection capability) in seasonally ice covered seas. Finally, additional efforts to better measure and parameterize surface exchanges in polar regions are much needed to improve coupled environmental prediction.

## INTRODUCTION

Over the last 10 years, there has been a significant maturing of ocean prediction systems, led by efforts such as GODAE OceanView (GOV; Bell et al., 2015; Davidson et al., 2019) and the European Union Copernicus Marine Environmental Monitoring Service (CMEMS; Le Traon et al., 2017). Numerous operational global and regional ocean analysis and forecast systems are now in place providing services for a range of applications including search and rescue, short- and long-range atmospheric and coupled prediction systems, aquaculture, energy sector activities and environmental management including environmental emergency response.

We have also seen the implementation of high-resolution operational ice-ocean prediction systems for polar regions providing forecasts on timescales of hours to days. These include the CMEMS Arctic Monitoring and Forecasting Centre (ARC MFC), the U.S. Navy Global Ocean Forecasting System, Environment and Climate Change Canada’s Global and Regional Ice Ocean Prediction Systems among others (Carrieres et al., 2017). While these systems are intended to provide support for marine operations and related applications, there has been a growing acceptance of the importance of including coupled interactions across the marine surface in numerical weather prediction systems (Brassington et al., 2015). Indeed, operational medium-range weather forecasting systems in Canada and at the European Centre for Medium Range Forecasting (ECWMF) now include a dynamic coupling with ice-ocean models (Mogensen et al., 2017; Smith et al., 2018). As a result, polar ocean observations may now have impacts beyond the polar regions on mid-latitude weather predictions (Jung et al., 2015). Moreover, coupled atmosphere-ice-ocean models have been shown to provide skillful forecasts at monthly-to-seasonal time scales in the polar regions, especially for the Arctic sea ice cover (e.g., Guémas et al., 2016a; Sigmond et al., 2016). The importance of polar sea ice initial conditions in affecting skill of monthly-to-seasonal predictions at lower latitudes in the atmosphere has also been discussed (e.g., Guémas et al., 2016b). These advances are also fostering a coupled modeling approach in the context of Earth system reanalyses for climate monitoring (e.g., Buizza et al., 2018).

Environmental prediction systems rely heavily on ocean observations from a variety of platforms (both *in situ* and from remote sensing). Indeed, Observing System Experiments (OSEs) have shown the delicate balance and complementarity provided by the current basket of observations (Oke et al., 2015; Fujii et al., 2019). However, polar regions present a number of unique observing challenges (see Calder et al., 2010). For short lead-times (hours to days), forecast skill depends strongly on initial conditions, which in turn depend on real-time observations ingested through data assimilation. The lack of real-time Argo profiling floats in polar regions due to the sea ice cover (Roemmich and Gilson, 2009) therefore creates a significant gap. This gap is compounded by additional errors in satellite products in polar regions, for example, due to difficulties in distinguishing snow and ice from clouds (Castro et al., 2016). The relative remoteness and harsh environmental conditions in polar regions further hinder efforts to provide *in situ* measurements. Moreover, the increasing use of coupled models noted above requires collocated observations of the atmosphere, ice and ocean, including flux estimates (Bourassa et al., 2013). This is all the more important because of the role of the Arctic region in particular in shaping the heat and freshwater transports at global scales, thus having a crucial remote impact on the mid- and low- latitude climate as well (Serreze and Barry, 2011; Jung et al., 2015).

Additionally, the rapidly receding summer ice cover in the Arctic is leading to both an increase in the demand for accurate ice-ocean and weather forecast products (Jung et al., 2016), as well as challenges in how to adapt prediction systems to accurately forecast conditions that may not have an existing analog in the historical record (e.g., the 2012 record low Arctic sea ice cover; Parkinson and Comiso, 2013). Similar issues apply in the Antarctic: rising temperatures and reports of both expanding and reduced sea ice have led to uncertainties about changes in the system (Maksym, 2019), while at the same time growing demands from Antarctic tourism and research logistics have driven a need for improved forecasts (Hoke et al., 2018).

Here, we outline the status of existing near real-time ocean observational efforts in polar regions, discuss gaps, and explore perspectives for the future. The first section is dedicated to *in situ* observations of temperature and salinity and several projects underway to further extend these efforts. Section “Satellite Observations” focuses on satellite observations of sea surface temperature (SST) and height (SSH), along with information on the sea ice cover. Section “Air-Sea Flux Measurements in Polar Oceans” discusses issues associated with surface flux measurements in polar regions. Section “Forecasting System Experiments” presents several impact studies in different prediction systems demonstrating the importance of ocean and sea ice observations to forecasting skill. Section “International Efforts to Address Gaps in Polar Ocean Observations” discusses several international projects working to address the gap in ocean observations in the polar regions. Finally, recommendations and an outlook for the future are presented in Sections “Recommendations” and “Outlook.”

## *IN SITU* OBSERVATIONS OF TEMPERATURE AND SALINITY

In the polar oceans there are several methods for collecting ocean and sea ice data and sending the data in near real-time via the Global Telecommunications System (GTS) or other transmission systems. In ice-free areas various methods are used, including ships, Argo floats, surface drifters, gliders, etc., while in ice-covered areas, only ice-borne instrument systems can operate throughout the year and transmit data in near real-time. Owing to their significant deployment and maintenance costs, the number of these platforms is very limited, resulting in large gaps in the data coverage in the ice-covered Arctic Ocean. Antarctic sea ice is largely single-year (or first-year) ice, which makes it ill-suited for ice based measurement systems. A significant fraction of the ocean data from the Arctic, as well as under-ice Argo profiles from the Antarctic seasonal ice zone, are only available in delayed mode, because underwater platforms in ice-covered areas cannot transmit data via satellite communication. This limits the access to ocean data available in near real-time, which is required by monitoring and forecasting services.

In the following section, the most common observing systems for the Arctic Ocean and Antarctic marginal sea are described briefly. Sections “The WHOI Ice-Tethered Profiler,” “ALAMO and Arctic Heat,” “Deployment of Argo Floats in Canadian Marginal Ice Zone by CONCEPTS,” “Under-ice BGC Argo Floats in the Canadian Arctic,” and “Surface Drifters and IABP” detail several specific observing efforts as exemplars of each class of technology.

### Overview of Current Observing System

#### Profiling Floats

Argo floats are the backbone of the global ocean observing system developed over the last two decades with more than 3500 units currently in operation (e.g., Roemmich et al., 2009). In the north, Argo floats are used in ice-free areas of the North Atlantic/Nordic Seas, Baffin Bay and Bering Sea. In the North Atlantic/Nordic Seas about 10–20 Argo floats are deployed each year sustaining an operational array of about 40 – 50 Argo floats in these regions. These floats are provided mainly by national efforts (e.g., NorArgo) and coordinated at the European level by EuroArgo. The funding of these Argo floats is relatively secure, and provided for by the countries that participate in EuroArgo.

There is ongoing development to adapt Argo floats to operate under ice, using ice-sensing algorithms as well as acoustic and optical techniques. In the Southern Ocean, the Argo program has demonstrated success with under-ice profiling floats that delay data transmission until the float is in open water, typically in summer (e.g., projects SOCLIM, RemOcean, SOCCO Bio-argo and ReMOCA). In particular, the Southern Ocean Carbon and Climate Observations and Modeling project (SOCCOM) has made an effort to deploy a significant number of underice Argo floats in the Southern Ocean. Moreover, RAFOS floats (Klatt et al., 2007; Reeve et al., 2016) provide the capability to triangulate their position using sound signals from nearby moorings for periods when they are not able to surface. In the Arctic, the ice-sensing algorithm is starting to become more mature and is being complemented with additional detection methods currently being tested by Takuvik (see Under-ice BGC Argo Floats in the Canadian Arctic).

#### Ice-Borne Observing Systems

Many types of ice-borne ocean measurement systems have been developed and fielded in the last 2–3 decades. Ice-based platforms are the only autonomous systems that can presently deliver near-real-time subsurface ocean observations year round from ice-covered areas. Early examples consisted of discrete sensors mounted on a floating mooring that drifts with the sea ice. More recent examples employ a drifting surface buoy deployed in the ice with a Conductivity-Temperature-Depth (CTD) sensor that is repeatedly transported along the mooring cable over the upper 800–1000 m of the water column. Ice-Tethered Profiler (ITP) systems from the Woods Hole Oceanographic Institution (WHOI, see The WHOI Ice-Tethered Profiler for details) have been fielded since 2004. Similarly, investigators from Laboratoire d’Océanographie et du Climat (LOCEAN) have developed and deployed IAOOS (Ice-Atmosphere-Arctic Ocean Observation System) buoys^1^. In addition to CTD profile observations, the IAOOS buoy includes ice mass balance measurements (snow and sea ice temperature and thickness), as well as a variety of meteorological sensors (Provost et al., 2015). Other similar platforms include the JAMSTEC Polar Ocean Profiling system (POPS; Kikuchi et al., 2007), the NPS Autonomous Ocean Flux Buoy^2^ (AOFB), and the UpTempO ice-tethered buoy developed by APL-UW (Castro et al., 2016). The Arctic University of Norway has recently developed and field tested a new platform called the Ice-Tethered Platform Cluster for Optical, Physical, and Ecological Sensors (ICE-POPE) that will be deployed operationally in 2019 (Berge et al., 2016). The Polar Research Institute of China is also developing ice-tethered platforms, which are being tested during Arctic expeditions of the I/B Xuelong. In comparison to Argo-type profiling floats, ice-based platforms are rather expensive (>100–300 k€ per unit). To date, funding to construct and field these systems and provide data to the community has derived from individual PI-led research projects. There are no operational programs to support the ice-tethered platforms at the moment, but there is a clear requirement from the Arctic Ocean modeling and data assimilation community that 10+ such platforms need to be deployed every year and supported operationally.

It is widely known that sea ice in the Arctic is shrinking in areal coverage, thinning, and becoming more mobile. All of these changes present complications to an ice-based observing system. Although diminished, the sea ice will remain critically important to earth’s climate and ecosystems as well as transportation and tourism, making ice-following observing platforms necessary into the future. However, future ice-borne instrument systems must be able to float and demonstrate resilience during fall freeze-up. Thinner, more mobile ice can be more prone to ridging that can damage ice based buoys. It has not proven feasible to maintain the array of 20 ice-based observatory systems in the Arctic that was envisioned at the turn of the century. Nor have these technologies been used extensively in the seasonal ice zone surrounding Antarctica, where sea ice typically melts completely every summer. Nevertheless, ice-based observatories have and are continuing to return valuable year-round upper ocean data from the central Arctic. Buoy clusters sampling various elements of the atmosphere, sea ice and upper ocean have proven particularly valuable.

#### Ice/Snow Surface Drifters

A reasonable number of low-cost ice buoys operate in the Arctic, providing ice motion, air pressure and surface temperature data. At present, most of these buoys are drifting in the western part of the Arctic, whereas the eastern part, including the Eurasian Basin and the Russian shelves, has very few buoys. The drifter data are transmitted via the Argos or Iridium satellite systems then posted on the WMO/IOC GTS and provide baseline data for weather and ice forecasting in the Arctic (see Section Surface Drifters and IABP for more detail). Drifters are also routinely deployed throughout the Southern Ocean, providing important information (e.g., SST and sea level pressure) to forecast systems from extremely data sparse regions.

Sea ice mass balance buoys (IMBs) provide information on temperature within snow and ice as well as ice thickness and its motion. Development and use of these buoys have continued for several decades with more than 100 IMBs deployed from 1957 to 2014. The data are transmitted in near realtime, but processing of the temperature profiles is mainly done manually. There are ongoing efforts to develop automated methods for retrieval of snow and ice thickness (Liao et al., 2018; Zuo et al., 2018). It is a challenge to develop robust algorithms that give accurate retrievals through the melting and freezing seasons.

Currently, three different systems are deployed in the Arctic including the CRREL-Dartmouth IMB (Richter-Menge et al., 2006), the SRSL Sea Ice Mass Balance Array (SIMBA; Jackson et al., 2013) and the TUT ice-tethered buoy developed by PRIC and TUC in China (Zuo et al., 2018). Some of the Ice-tethered platforms described in Section “Ice-Borne Observing Systems” are also equipped with SIMBA instruments.

#### Other Systems

1. Ferrybox systems: The Norwegian Institute for Water Research (NIVA) operates a ferrybox between Tromsø and Longyearbyen/Ny Ålesund. Data are obtained every 2 weeks year-round. This system is fairly sustainable through support from a new infrastructure project led by NIVA.
2. Ship-based CTD data during scientific cruises and fishery management cruises: Several research vessels in the North Atlantic/Nordic seas deliver CTD observations in near real-time. The data are provided more or less regularly, depending on the schedule of the research vessels.
3. Profiling gliders: Several institutions conduct ocean glider experiments in the Arctic (e.g., Nordic Seas, David Strait, Fram Strait, north of Svalbard, Beaufort Sea) providing CTD data in near real-time. These experiments are mainly in the summer season, however, acoustically navigated ice-capable Seagliders (developed by APL-UW) were successfully used for the year-round missions in Davis Strait and in the Beaufort Sea including under ice measurements in winter (Curry et al., 2014; Lee et al., 2016). Glider experiments are also conducted with growing frequency in the Southern Ocean. These provide valuable data and upper ocean process understanding from winter through summer (e.g., Swart et al., 2015; Thomson and Girton, 2017; du Plessis et al., 2019 – in review).
4. Autonomous surface vehicles: Wave Gliders have been deployed in both the Arctic and Southern Ocean, providing rare surface flux (heat, momentum and CO_2_) information over multiple months at a time and with high resolution (Monteiro et al., 2015; Schmidt et al., 2017; Thomson and Girton, 2017). Many of these platforms are reporting or being adapted to report real-time surface observational data.
5. Sea-mammal borne instruments (CTD data) are becoming the most numerous *in situ* observations at high latitude in the Antarctic and also more recently in the Arctic, and may improve forecast skill and circulation patterns (Fedak, 2013; Roquet et al., 2013; Carse et al., 2015).
6. Acoustic tomography: Acoustic sources and receivers have been deployed in the Arctic in the past (Mikhalevsky et al., 2015) and offer the unique capability to measure large-scale changes in temperature and heat content of the Arctic Ocean over long periods (Howe et al., 2019). New deployments of this technology were conducted under the CANAPE project (led by Scripps); CAATEx programs (led by NERSC) have been approved. While no observations are currently available in real-time, nor assimilated in delayed mode, acoustic tomography nonetheless presents an interesting possibility for constraining ocean state-estimate/prediction systems.
7. Moored Arrays: Several long-term observatories have been running in both ice-covered and open-water areas with the capacity to provide real-time data. Examples include the German long-term observatory FRAM (Frontiers in Arctic Marine Monitoring; Soltwedel et al., 2013) and the Barrow Strait Observatory (Richards et al., 2017).

### The WHOI Ice-Tethered Profiler

**FIGURE 1 ∣:**
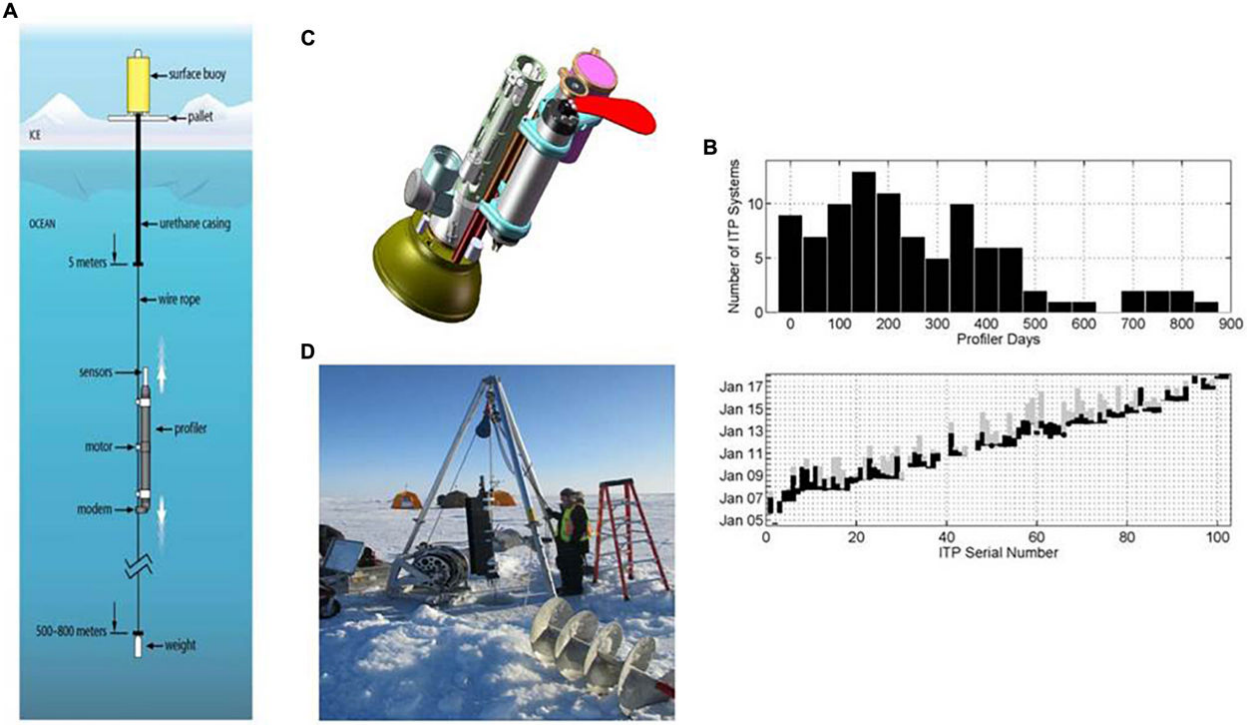
**(A)** Schematic drawing of the WHOI Ice-Tethered Profiler (ITP) system; **(B)** Histogram of ITP underwater vehicle lifetimes (top) and (bottom) the periods (shown as black vertical bars) over which telemetry was received from each ITP underwater unit and from each corresponding surface buoy (black plus gray bars). The history of ITP systems deployed in the Southern Ocean and in lakes are excluded from this plot. **(C)** Schematic drawing of the bio-optical ITP sensor suite with CTD/O_2_, chlorophyll fluorescence, CDOM, optical backscatter and PAR (the latter suite housed under a retractable shutter), and **(D)** installation photograph of an ITP with a Modular Acoustic Velocity Sensor (ITP-V).

Historically, the Arctic Ocean has been poorly sampled relative to waters at low- and mid-latitudes, especially in winter time. To address this observing shortfall, the WHOI ITP was designed to sample the upper ocean below drifting sea ice throughout the year and to return data in near real time (Krishfield et al., 2008; Toole et al., 2011). As noted earlier, there are now a variety of systems with similar capability now being fielded; the ITP is detailed here as exemplar of the group. The expendable ITP consists of a surface buoy that supports a weighted wire-rope tether extending through the ice and down to (at most) 800 m depth (Figures 1A,C,D). The heart of the ITP system is a cylindrical vehicle fitted with sensors (similar in size and shape to an Argo float) that travels up and down the tether. ITPs are equipped with CTD instruments for observing the ocean’s thermohaline stratification and may also include sensors that sample, for example, dissolved oxygen (Timmermans et al., 2010), bio-optical properties (Laney et al., 2014), and ocean currents (Ice-Tethered Profiler with Velocity – ITV-V: Thwaites et al., 2011; Cole et al., 2015). In addition, instruments measuring temperature-conductivity, pCO_2_, dissolved O_2_ and pH have been affixed to the tether above the profiling interval (e.g., Islam et al., 2017). Deployments may be done from ice camps (supported by fixed-wing aircraft or helicopters) or ships. The majority of deployments have been through holes augured through ice floes but a handful of systems have been installed in open water (the buoy has sufficient buoyancy to support the system); most of those have survived fall freeze-up.

The basic ITP system was designed for an operational lifetime of more than 2 years assuming approximately 1500 m of profiling per day (e.g., 2 one-way profiles of 750 m span). Actual lifetimes of the full ITP system are often less than this, Figure 1B. There are two major failure modes of ITPs: crushing of the surface buoy and/or breaking of the tether in ice ridging events and dragging of the tether in shallow water. As is evident in Figure 1B, ITP surface buoys frequently transmit position data for an extended time after communication with the underwater unit is lost.

ITP data, available from the project websit^3^, the National Environmental Data Center and the Arctic Data Center, support a range of scientific investigations and student projects. The basin-wide and year-round coverage facilitates studies of seasonal-to-interannual physical and biogeochemical processes (e.g., Rabe et al., 2011; McPhee, 2013; Laney et al., 2014, 2017; Islam et al., 2017) and basin-scale phenomena (e.g., Timmermans et al., 2014), as well as supporting the initialization (data assimilation) and/or validation of numerical models. Smaller-scale processes may also be investigated with ITP data, including meso- and sub-mesoscale variability (e.g., Timmermans et al., 2011; Zhao et al., 2014, 2016), near-inertial internal waves (Cole et al., 2014; Dosser et al., 2014) and double diffusion (e.g., Timmermans et al., 2008; Shibley et al., 2017). Notably, the range of sensors able to be supported on ITPs provides a wide-ranging view of the evolving Arctic Ocean system.

### ALAMO and Arctic Heat

Since 2014, NOAA Pacific Marine Environmental Laboratory (PMEL) and the University of Washington’s Joint Institute for the Study of the Atmosphere and Ocean (JISAO) have been developing and field testing a series of autonomous marine systems designed for Arctic applications. This work has been carried out in collaboration with scientists, forecasters, and industry, primarily under the auspices of the Innovative Technologies for Arctic Exploration (ITAE^4^) program. Fifteen new types of technology have been developed or advanced for Arctic use under this program, including the Air-Launched Autonomous Micro-Observer (ALAMO), the Saildrone autonomous marine vehicle, and the Oculus Coastal Glider.

**FIGURE 2 ∣:**
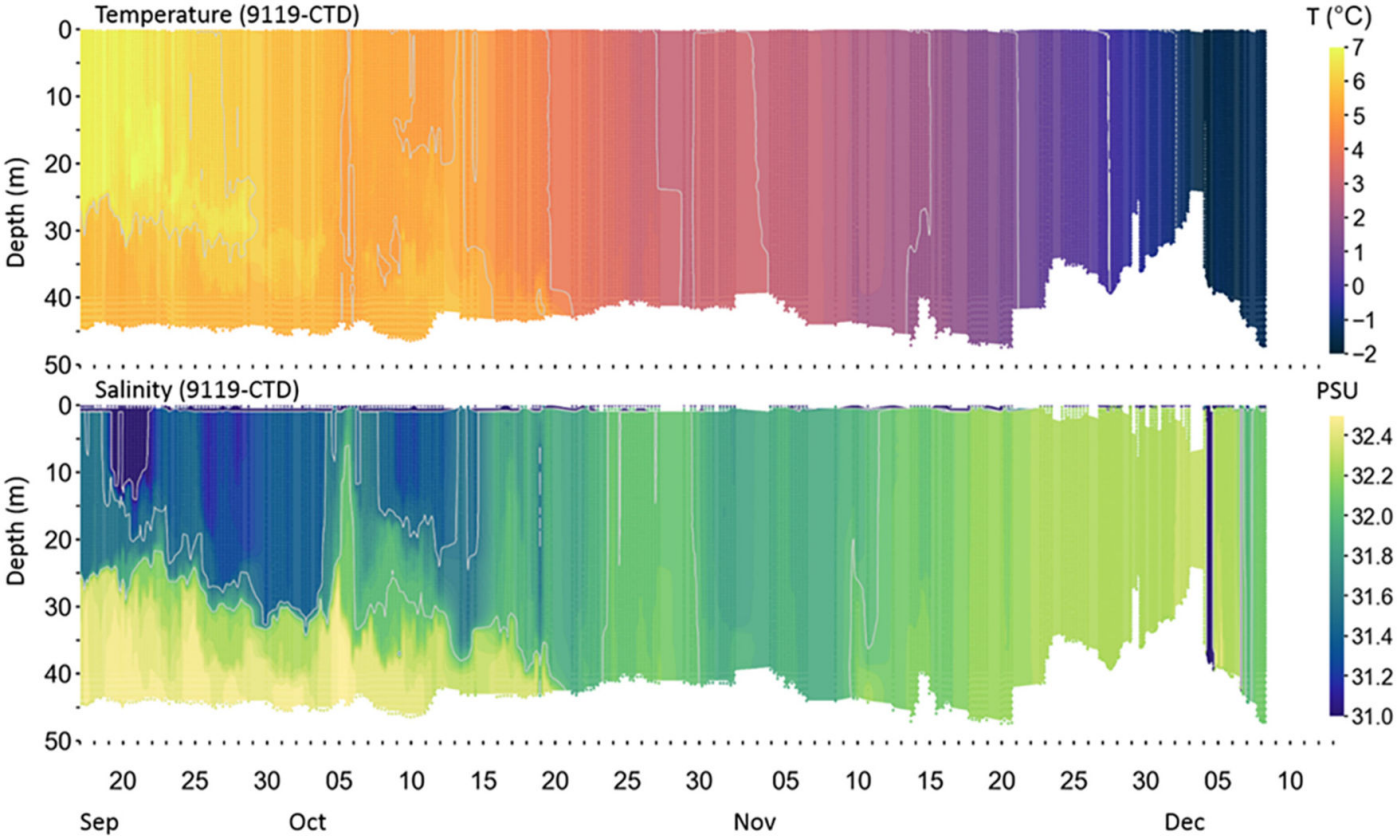
Temperature and salinity profiles collected by ALAMO 9119-CTD from September 17 to December 8, 2017. The float began sampling near 167W, 70N and the last profile was near 165W, 72N. See: https://www.pmel.noaa.gov/arctic-heat/ for more information, including the float track.

An example of a multi-platform experiment along these lines is the Arctic Heat Open Science Experiment (Wood et al., 2018). Here a NOAA Twin Otter research aircraft (NOAA-56) has been equipped with a range of weather and ocean-sensing instruments, which in 2018 included flight-level weather and radiometry, an A-size sonobuoy deployment tube used for a range of air-deployed probes – including AXBT/AXCTD/AXCP, atmospheric dropsondes, ALAMO autonomous profilers, and experimental UAS (Unmanned Aerial Systems) platforms – LIDAR and thermal imaging camera. Arctic Heat deploys an array of ALAMO floats in the Chukchi Sea between May and September; floats deployed later in the season are active into winter (Figure 2), and are used to develop an experimental seasonal freeze-up projection, in combination with satellite-derived SST and historical sea ice extent/concentration. In 2018 a combination of more than two dozen profiling floats of various types were deployed by three collaborating research groups in the Chukchi and Beaufort Seas^5^.

The dense array of floats in 2018 provides an opportunity to thoroughly investigate the potential gain in predictive skill provided by enhanced real-time ocean observations. Understanding how assimilating more observations will impact modeled analyses and short-term forecasts is of fundamental importance as new coupled models are developed. For example, sensitivity experiments with the NOAA-Earth System Research Laboratory Coupled Arctic Forecast System (CAFS) (see NOAA-ESRL/CIRES Coupled Arctic Forecast System) are being pursued.

## Deployment of Argo Floats in Canadian Marginal Ice Zone by CONCEPTS

A Government of Canada initiative called CONCEPTS (Canadian Operational Network of Coupled Environmental Prediction Systems; Davidson et al., 2013) has implemented global and regional environmental prediction systems over the last few years. A particular need has been identified to complement the existing observing system with additional real-time observations to better constrain water mass properties in the ocean analyses, in particular in Canadian seasonally ice-infested seas. As a result, a project is underway to test the implementation of different observing technologies for their ability to provide reliable measurements of ocean temperature and salinity at a low cost. A first step was the deployment of a standard Argo float in the Gulf of St. Lawrence, configured with a shallow profiling and parking depth (200 m). The objective was to provide observations over the spring-to-fall ice-free period and to allow the float to remain subsurface in winter when ice forms. The use of existing technology (i.e., without any particular ice detection capability) reduced costs and increased feasibility. This initial effort was quite successful and provided an excellent complement to existing (and more costly) moorings deployed in the Gulf of St. Lawrence.

**FIGURE 3 ∣:**
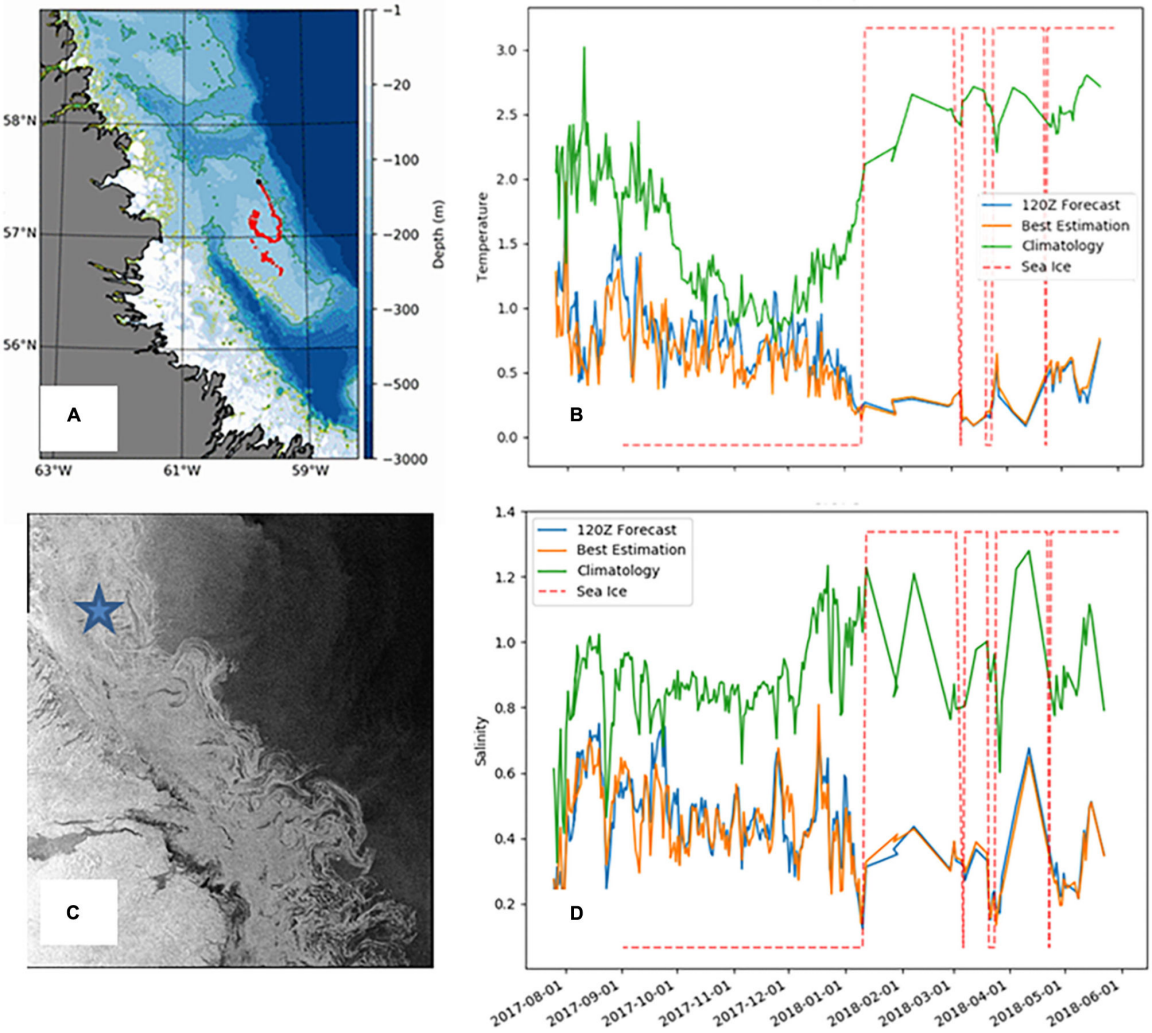
Measurements from an Argo float deployed on the Labrador Shelf. **(A)** Show the location of transmissions from the float over the period 01-August-2017 to 01-June-2018. **(C)** Show a Synthetic Aperture Radar image from RADARSAT-2 for 28-Jan-2018 with a blue star indicating the location of the Argo float. **(B,D)** Present analyses (orange) and 5-day forecasts (blue) from the Global Ice Ocean Prediction System for temperature and salinity respectively. Also shown are values from the World Ocean Atlas 2013 climatology (green). The presence of sea ice is indicated by the dashed red line, with values near the bottom indicating no ice and values near the top of the panel indicating the likely presence of ice as detected by GIOPS ice analyses.

A second effort included the deployment of an Argo float on the Labrador shelf, again with a shallow profiling depth of 200 m. This depth was greater than the local bathymetry allowing the float to rest on the sea floor and reduce lateral drift. Despite the presence of ice in winter, this float was able to breach the surface periodically (roughly every 10 days) and transmit ocean measurements. Additionally, the float observed temperature and salinity measurements during winter that deviated significantly from climatological conditions (Figure 3). As these climatological values are typically used by various applications in the absence of other data, the Argo float observations filled an important gap. Moreover, their availability in real time permits their use in data assimilation systems that can allow the detected anomaly in water mass conditions to be propagated over suitably correlated water masses (i.e., along the Labrador Shelf).

As part of the YOPP (see Year of Polar Prediction), this effort has been expanded to include the deployment of 7 Argo floats during the Arctic summer Special Observing Period (July–September 2018). If successful, and found to provide adequate benefit for the cost, this effort could become part of the Canadian Argo Program, and also contribute to the newly forming Canadian Integrated Ocean Observing System (CIOOS). The use of air deployable ALAMO floats and floats with ice-detection capabilities is also being investigated.

### Under-Ice BGC Argo Floats in the Canadian Arctic

**FIGURE 4 ∣:**
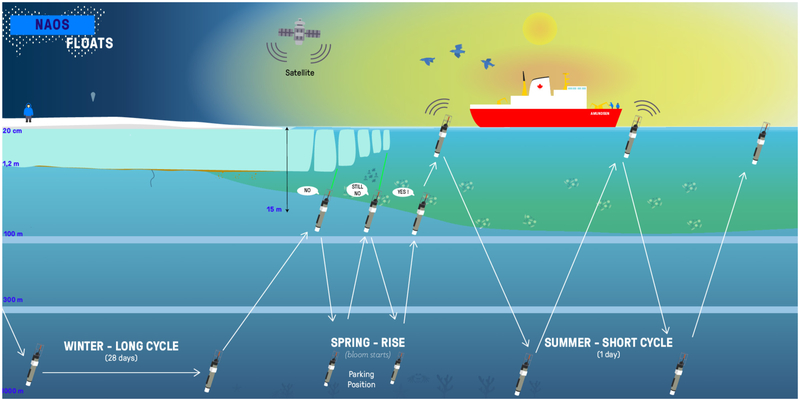
A daily profile cycle of BGC-Argo floats deployed during the 2016 Green Edge scientific mission in Baffin Bay. The main goal is the understanding of the dynamics of the phytoplankton spring bloom and determine its role in the Arctic. During the spring-period the risk of colliding with sea-ice when emerging is a threat to the security of the floats. Moreover, during wintertime, geo-localization and the use satellite networks for data transmission and commands reception is not yet possible (Credit: J. Sansoulet, Takuvik).

As part of project Green Edge^6^, Takuvik deploys Biogeochemical Argo floats (BGC Argo; manufactured by NKE^7^) in the Arctic Ocean to study the dynamics of ice-edge phytoplankton spring blooms as controlled by sea ice dynamics, vertical mixing, light and nutrients. BGC Argo floats are more specialized profilers, equipped with sensors capable of sampling additional essential ocean variables (Claustre et al., 2010). The study focuses on Baffin Bay, which involves navigational challenges for floats in terms of bathymetry, ice coverage and circulation. Simulations of trajectories to choose the best dropping zones, combined with observations from ice charts (climatology and real-time charts) are necessary for safe deployments. An ice-covered ocean presents a real challenge for Argo floats that must surface for geo-localization and to use satellite networks for data transmission and command reception (Riser et al., 2016), see Figure 4. Therefore, some technical refinements have been needed to make the floats operational in the Arctic Ocean (Fennel and Greenan, 2017).

The development of a new generation of floats (PRO-ICE) to be operated under ice, was founded by the French project NAOS^8^. If sea ice is present at the surface, Argo floats need to postpone surfacing. It will perform several consecutive profiles without sending stored data. PRO-ICE have additional nonvolatile memory and are able to record data from all profiles including those performed during wintertime. Because of the need to transmit a high volume of data, communications are performed via a two-way Iridium link, which is faster than the older ARGOS link. This minimizes time at the surface and allows instructions to be transmitted to the float. In addition, floats to be deployed in Arctic seas need to be able to deal with a wide range of seawater salinity/density. Some Argo floats, like PRO-ICE, do not require pre-ballasting as a function of mean seawater density. This is a huge advantage since they can surface easily in a low-density surface seawater layer (Riser et al., 2016).

The PRO-ICE floats use a combination of three technologies to detect ice: a reversed altimeter (active acoustics), an Ice Sensing Algorithm (Klatt et al., 2007) (ISA) based on seawater freezing temperature and an optical sensor. In the Arctic Ocean, the threshold of the ISA algorithm is difficult to determine because of the high levels of variation in salinity (seasonal and regional), compared to the homogeneous salinity present in Antarctica. Takuvik gathered a substantial data base of temperature and salinity data in Baffin Bay, linked to ice cover information in order to locally adapt the threshold of ISA.

Furthermore, the resolution provided by an altimeter is not sufficient to detect a thin-ice layer. For this, Takuvik has developed an ice-detection system based on laser polarimetry (Lagunas et al., 2018). Since sea-ice is a strong light depolarizer, this characteristic was used as an indicator of the presence or absence of ice. This ice-detection system has been installed on several PRO-ICE BGC-Argo floats deployed yearly in Baffin Bay since 2016.

A potential solution to data transmission issues caused by an inability to surface under the ice cover may be provided by underwater acoustic networks. Multipurpose underwater acoustic networks can provide under-ice positioning (“underwater GPS”; Lee and Gobat, 2008) and low bit-rate communication services (Freitag et al., 2015) to AUVs fitted with low-power acoustic receivers. Acoustic positioning systems have been deployed for under-ice navigation of Argo floats in the Weddell Sea (Klatt et al., 2007). Challenges for designing basin-scale acoustic systems include modeling the time-varying nature of the Arctic sound channel, for which measurements of annual variations of under-ice sound profiles and ice-bottom roughness are scarce (Rehm et al., 2018). The role of multipurpose acoustics networks as key components of polar observing systems is explored further in a complementary article (Howe et al., 2019).

Data acquired by Takuvik’s PRO-ICE floats are available in the Argo database at the CORIOLIS Global Data Assembly Centre^9^ (GDAC). The data policy set for Argo mandates free access to the data for all interested scientists, research groups and operational agencies. The data management is designed to facilitate easy and immediate access to data not only in real time, but also in delayed mode.

### Surface Drifters and IABP

**FIGURE 5 ∣:**
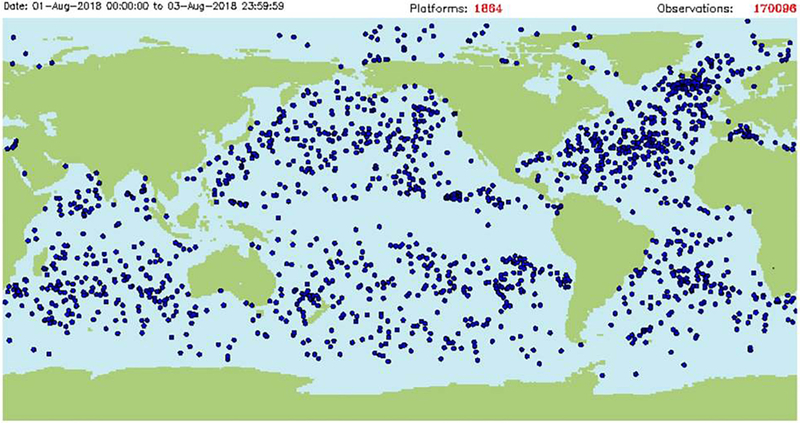
Map of drifting buoys reporting on the WMO/IOC GTS in August 2018. Source: http://OSMC.NOAA.GOV.

**FIGURE 6 ∣:**
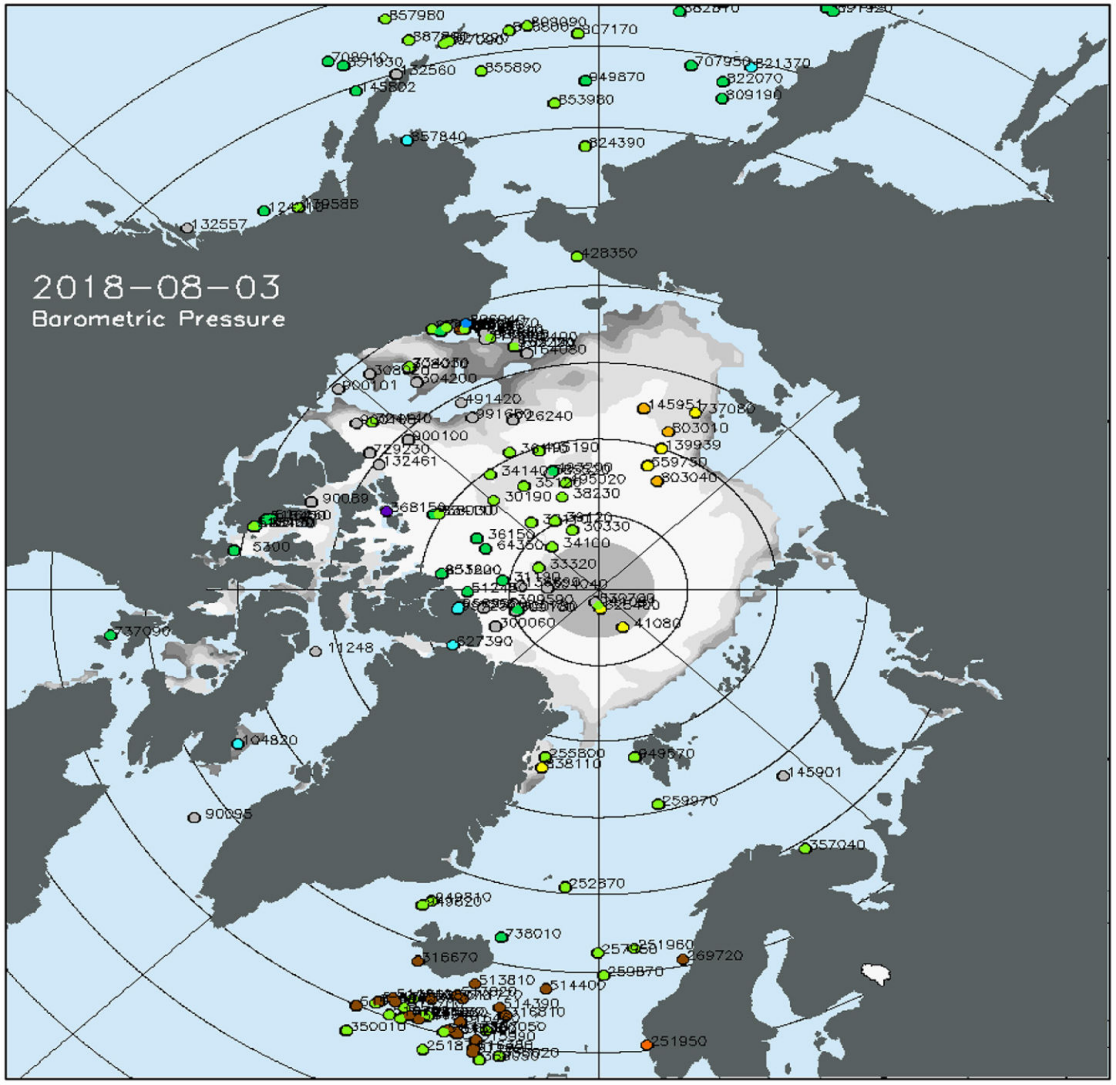
Map of drifting buoys reporting in the Arctic on August 3, 2018. Source: http://IABP.apl.uw.edu.

Surface air pressure, temperature and ocean/ice circulation in the polar regions (Figure 5) are observed by a network of drifting buoys maintained by participants of the International Arctic Buoy Programme (IABP^10^, Figure 6) and the International Programme for Antarctic Buoys (IPAB^11^). Over the Arctic Ocean and its peripheral seas (north of 60°N), there were about 120 buoys reporting as of August 2018. Most of these buoys were located in the North American sector, with only a handful reporting in the Eurasian sector of the Arctic. This gap in the Arctic Observing Network creates a significant uncertainty in the analyzed fields of sea level pressure, temperature and winds (Inoue et al., 2009, Figure 7).

In the Southern Ocean (south of 40°S) there were about 50 buoys as of August, 2018. Although over 100 buoys were deployed during the Austral summer near the coast of Antarctica, most of these buoys were destroyed by the sea ice during freeze up or have been blown north, away from the coast by the prevailing winds.

Maintaining the polar observing networks near the coasts around most of Antarctica, and in the Eurasian Arctic is an ongoing challenge, since the prevailing winds and ocean currents quickly transport the buoys away from the coast. Ideally, the IABP and IPAB networks should be reseeded during the winter, but it is difficult to deploy buoys from ships and aircraft during the polar night and extreme cold and harsh conditions of winter.

The participants of the IABP and IPAB strive to release their data onto the WMO/IOC GTS in near real-time by both the global research and operational weather and ice forecasting communities.

## SATELLITE OBSERVATIONS

Satellite observations of polar oceans have been acquired for more than four decades by different measuring systems. The most prominent record comes from microwave radiometers that have been documenting the reduction of sea ice extent (Cavalieri and Parkinson, 2012; Osborne et al., 2018). Remote sensing systems also provide valuable information about other parameters of the sea ice (concentration and thickness) or about the underlying ocean (temperature and circulation). However, despite continuous technological advances and progress in data processing, several weaknesses can be pointed out and should be considered in the future for improving the observing system of polar regions.

### Sea Ice Concentration

Satellite-based sea ice observations are required for assimilation to provide accurate polar environmental forecasts. These observations have been proven beneficial in improving prediction skill at different temporal scales when assimilated (e.g., Lisaeter et al., 2003; Lindsay and Zhang, 2006; Stark et al., 2008; Massonnet et al., 2013; Tietsche et al., 2013). In addition, it has also been shown that summer sea ice thickness (SIT) can be constrained to some extent by assimilating sea ice concentration (e.g., Yang et al., 2015). However, there is a significant spread in sea ice concentration products obtained through different retrieval algorithms (Ivanova et al., 2014), which affects the consistency of ocean-sea ice analyses that assimilate those products (Chevallier et al., 2016; Uotila et al., 2018), and the skill of seasonal predictions initialized from those reanalyses (e.g., Bunzel et al., 2016).

### Sea Ice Freeboard and Thickness

Observing SIT from space is a challenge (Kwok and Sulsky, 2010), and gridded data are sparse. Recently SIT has been estimated using altimetry (CryoSat-2; Kwok and Cunningham, 2015), or the L-band radiometers of SMOS (Kaleschke et al., 2012; Tian-Kunze et al., 2014) and NASA’s SMAP (Patilea et al., 2019) satellites, for thinner ice. The ICESat-2 laser altimeter satellite, which was launched in September 2018, is also starting to provide polar SIT estimates.

Sea ice thickness is typically derived from radar altimetry observations of freeboard using the hydro-static equilibrium assumption (e.g., Alexandrov et al., 2010). Both the freeboard retrieval from altimeter measurements (e.g., Ricker et al., 2014) and the freeboard to thickness conversion are active fields of research. The freeboard to thickness conversion is constrained by the limited availability of auxiliary information (sea ice type, density and snow parameters). Snow thickness on sea ice is still poorly known and is a major source of uncertainty. Snow depth models are not yet satisfactory, and alternative strategies will need to be defined to permit improving snow thickness estimation over sea ice. The joint use of Ku and Ka frequencies may facilitate estimates of the snow load above the ice pack. Studies based on AltiKa (Ka-band about 35.7 GHz) and CryoSat-2 (Ku-band about 13.5 GHz) satellite data have shown that differences in penetration of Ka- and Ku-band are correlated with snow loading on sea ice (Armitage and Ridout, 2015; Guerreiro et al., 2016). Although AltiKa only provides measurements up to 81.5°N and thus cannot provide pan-Arctic data. The combination of laser (ICESat-2) and radar altimetry is also promising for this purpose. A better estimation of freeboard and then thickness would greatly benefit from such measurement complementarity. Data editing is also important on heterogeneous surfaces such as sea ice, where melt ponds act as bright targets in the radar footprint resulting in peaky waveforms that look very similar to returns from leads. As a result, radar altimeter ice thickness products based on CryoSat-2 (e.g., Hendricks et al., 2016) are not available in summer months.

Previous studies showed that assimilation of SMOS ice thickness significantly improves the first-year ice estimates (Yang et al., 2014; Xie et al., 2016). Furthermore, assimilating CryoSat-2 and SMOS SIT leads to a reliable pan-Arctic SIT estimates (Mu et al., 2018; Xie et al., 2018), and also has the potential to improve seasonal forecasts of Arctic sea ice (Chen et al., 2017; Blockley and Peterson, 2018). The Cryorad mission has been proposed by Macelloni et al. (2017) as a microwave radiometer at very low frequency (down to 500 MHz, lower than SMOS) which longer wavelengths would penetrate through thicker SIT. A critical difficulty related to this mission is the contamination by Radio-Frequency Interference.

### Sea Surface Temperature

Satellite SST retrievals in polar regions are challenging. SST is estimated from instruments operating in the infrared (IR) and microwave regions of the spectrum through the so-called atmospheric window regions. IR retrievals in polar regions are often limited by the observed abnormal (often very dry) atmospheric conditions (Vincent et al., 2008). In addition, there are issues in detecting clear-sky ocean conditions that are free from cloud and sea ice, issues which are compounded during polar nights and in areas of persistent cloud. Consequently, frequent measurements of SST at high latitudes rely on microwave imaging instruments. Although not impacted by cloud (unless precipitation), microwave retrievals are impacted by issues in detecting sea ice, especially at the edges of the instrument footprint.

Many SST retrieval algorithms for both IR and microwave rely on *in situ* data to account for both deficiencies in their calibration as well as correcting for atmospheric attenuation. The lack of *in situ* data should be addressed and new innovative approaches are needed (e.g., Castro et al., 2016). The Group for High Resolution Sea Surface Temperature (GHRSST) coordinates the production of multi-satellite merged SST products. However, the lack of accurate satellite and *in situ* data means there is little consistency between products, especially at high latitudes (Dash et al., 2010).

The current lack of continuity of microwave imagers that can be used to derive global SST is a major concern. For polar regions this requires the inclusion of a channel around 6.9 GHz (Gentemann et al., 2010). Currently, the only approved future instrument with this capability is the Chinese Microwave Radiometer Imager (MWRI) onboard the HaiYang-2B (HY-2B). A Copernicus Imaging Microwave Radiometry (CIMR) is currently being studied by the European Space agency (ESA) and JAXA is planning a follow-on to the Advanced Microwave Scanning Radiometer (AMSR2). The AMSR3 and CIMR missions are highly complementary and in combination would provide improved coverage and sampling in polar regions.

### Sea Surface Height

**FIGURE 8 ∣:**
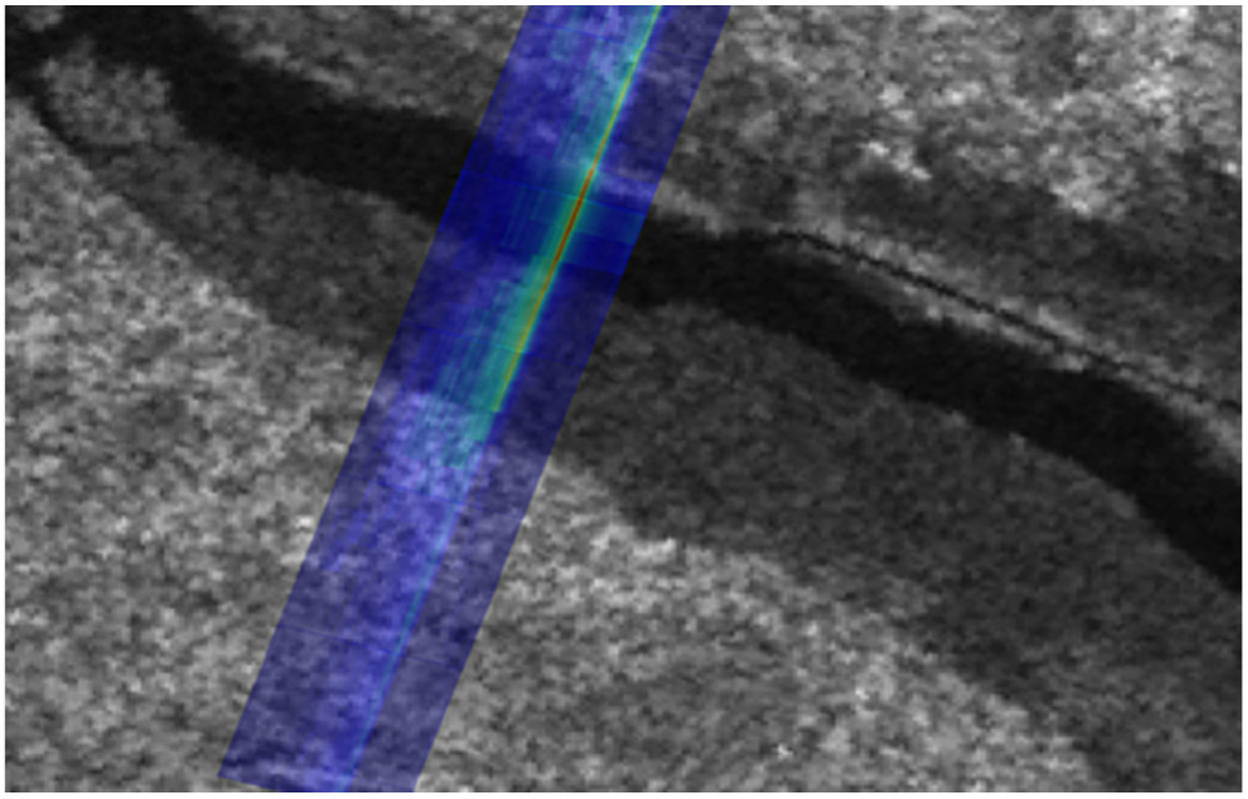
Collocation of one Sentinel-1 SAR image (background) and Sentinel-3 altimeter waveforms (Unfocused processing; color) over a lead in the Arctic Ocean.

Satellite observations of sea level are required to constrain surface geostrophic currents in ocean forecasting systems, and several teams try to tackle the issue (Prandi et al., 2012; Andersen and Piccioni, 2016; Armitage et al., 2016). However, sea level observations from altimetry over polar regions suffer from three main issues:

1. First, the altimeter constellation has mainly been created to fulfill ocean requirements for the ice-free regions, in particular with regard to orbit coverage/inclination. With the exception of the CryoSat-2 mission, which covers the Arctic Ocean up to 88°N, altimetry missions do not cover poleward of 82°, leaving a vast region without any measurement.
2. Second, although significant progress has been made to distinguish whether measurements correspond to open ocean, ice floes or leads within the sea ice, further progress is still needed to unambiguously identify the different returns, in particular in complex mixed water/floe areas. Exploitation of close match-ups between SAR imagers and altimeter measurements as the potential for improving the identification of leads and the editing of ambiguous measurements coming from melt ponds or polynya in the melt season. An example of such collocation between a Sentinel-1 image and Sentinel-3 measurement is provided in Figure 8 (Longépé et al., 2019).
3. Lastly, the accurate retrieval of absolute SSHs, and therefore currents, in polar regions also suffers from degraded corrections. Tide models show higher inter-model variability in polar regions than anywhere else, mean sea surfaces are not as accurate. An effort to refine the geophysical corrections applied to altimeter measurements is needed to improve polar SSH accuracy levels and make them useful for assimilation in operational oceanography systems.

As a result, there is currently no dedicated operational Arctic sea level product for assimilation in models. Efforts to provide Arctic sea level information in CMEMS, following the current SL-TAC products are ongoing.

Other innovative concepts such as SKIM’s (Sea surface Kinematics Multiscale monitoring, Ardhuin et al., 2018) rotating radars at different angles offer opportunities to monitor waves, surface currents and possibly sea ice drift. The Surface Water Ocean Topography (SWOT) mission also has the potential to provide innovative new observations for sea level and sea ice cover, although its orbit is lower than 70 degrees.

### Sea-Ice Drift

Sea ice drift data are now obtained all year round both in the Arctic and Antarctic by pattern cross-correlation of scatterometers and passive microwave images. For global information, Scanning Multichannel Microwave Radiometer (SMMR), the Special Sensor Microwave/Imager (SSM/I) and SSMI/Sounder (SSMIS), and the Advanced Very-High-Resolution Radiometer (AVHRR) are used. Currently, daily products (e.g., Lavergne et al., 2010; Tschudi et al., 2019) typically use data acquired at Day D-1, with span of 24 or 48 h. In addition, buoy observations of the International Arctic Buoy Program (IABP), and ice motion derived from NCEP/NCAR surface wind vectors can be used. Low resolution ice drift products are calculated daily from aggregate charts derived from radiometers (e.g., SSMIS, AMSR2) or scatterometers (e.g., ASCAT). The typical resolution of these input images is 12.5 km. The large acquisitions, the repetition of the acquisitions, and their independence with respect to the weather conditions allow a daily polar coverage.

Sequences of SAR images can be used to derive higher resolution drift information. Algorithms have been developed to calculate ice drift from successive pairs of SAR images covering a common area. They are generally based on a spatial correlation calculation between these images, at several resolution scales (from the coarsest to the finest). In Europe, Sentinel-1 is used by DMI to produce an operational sea ice drift product as part of CMEMS (Pedersen et al., 2015). SAR data from Sentinel-1 A/B constellation allow the derivation of daily fields of sea ice deformation at 2 km resolution (Korosov and Rampal, 2017). The algorithm developed by FMI has been operational in the Baltic Sea since early 2011 (Karvonen, 2012), using the wide Radarsat-1 ScanSAR mode and the wide swath ASAR Envisat mode data.

Revisit is the key here: higher revisit of SAR images is naturally required. Algorithms often use image tracking features between consecutive images. This type of algorithms perform better if the images are taken with the same frequency, short interval, and ideally same geometry. Joint acquisition of multifrequency SAR would enable accurate sea ice drift products, which is not possible with stand-alone current mono-frequency SAR missions. Cross-pol channel is often preferred as it is less sensitive to incidence angle variation (at least for C-band SAR). Drift cannot be calculated in areas without characteristics (i.e., so-called "level ice" and open water areas). These are of course only estimates of the integrated ice trajectories between the time instants corresponding to the SAR acquisition times. Lagrangian drift products are typically 2-day trajectories with coarse resolution (62.5 km) and are not often assimilated even though the accuracy is satisfactory (3 km for 2-days drift). The TOPAZ4 system does assimilate these sea ice drift operationally, although the effect is relatively weak (Sakov et al., 2012). A different sea ice rheological model, more sensitive to winds, may be more adapted to assimilate this data (Rabatel et al., 2018). More precise (500 m for 1-day drift) and detailed (10 km resolution) sea ice drift products are now obtained year-round from Sentinel-1 SAR images, which cover about 70% of the Arctic as of February 2019.

## AIR-SEA FLUX MEASUREMENTS IN POLAR OCEANS

### The Need and Challenge for Air-Sea Flux Observations in Polar Regions

Air-sea fluxes quantify the exchanges of heat, momentum, freshwater, gases and aerosols between different components of the polar climate system (i.e., atmosphere, water column, sea ice). Flux observations are essential for understanding the global energy budget (Trenberth and Fasullo, 2010), for evaluating forecasting and climate models, and for evaluating processes such as ocean heat uptake and mixed-layer temperature and SST variability (e.g., Dong et al., 2007). However, *in situ* air-sea or air-ice flux observations are extremely sparse in polar regions (e.g., Bourassa et al., 2013), with almost no winter observations in the Southern Ocean (e.g., Gille et al., 2016; Swart et al., 2019) or in ice-covered ocean domains in both the Arctic and Antarctic (e.g., Taylor et al., 2018). Quantifying air-sea exchange in regions with sea ice requires specialized approaches that must account for spatio-temporal heterogeneity. This has led to significant gaps in our knowledge of both air-sea and air-sea-ice exchanges.

Since there are few reliable near-surface atmospheric observations to serve as constraints, reanalyses and satellite derived surface flux products have major errors and vary considerably between products (Josey et al., 2013; Bentamy et al., 2017; Schmidt et al., 2017; Taylor et al., 2018). For example, in the Southern Ocean, atmospheric models (and reanalyses) have large air-sea heat flux biases, including substantial short-wave errors related to their inability to adequately represent super-cooled liquid cloud water (Bodas-Salcedo et al., 2014, 2016), and these errors appear to bias coupled model SST (Hyder et al., 2018), sea ice, and wind (Bracegirdle et al., 2018). Moored-buoy flux observations or year-round ice camps are required to evaluate and ultimately improve these products but to date buoys have been deployed in only two locations in the Southern Ocean (Schulz et al., 2012; Ogle et al., 2018) and in only a few instances in the Arctic, (Taylor et al., 2018). The Surface Heat Budget for the Arctic Ocean (SHEBA) program offered the only year-round ice camp in the Arctic (e.g., Persson et al., 2002), although Multidisciplinary drifting Observatory for the Study of Arctic Climate (MOSAiC; see MOSAiC) will soon extend this. The Southern Ocean Observing System (SOOS) working group on Southern Ocean air-sea fluxes (SOFLUX; Swart et al., 2019) has been working to coordinate observing system capabilities and requirements for high-latitude air-sea fluxes.

### Impact of Flux Uncertainty on Forecasting/Prediction

There are significant advantages to forecasting with a coupled atmosphere-ice-ocean system, especially on longer timescales. The intrinsic turbulence of the atmosphere limits predictive skill to timescales of days to weeks (Mariotti et al., 2018), whereas the large-scale ocean is predictable on monthly time scales. This provides a mechanism by which the predictability of atmosphere may be extended allowing skillful seasonal predictions.

A particular limitation in this regard is with respect to the significant uncertainty in the atmosphere-ocean boundary layer, made even more egregious when considering sea ice. Boundary layer dynamics involve vertical scales unresolved by coupled forecasting systems, which must be parameterized (e.g., Pullen et al., 2017). The exchanges between components are parameterized by so-called bulk formulae, which estimate air-sea-ice exchanges based on near-surface large-scale properties. Currently, the uncertainties in bulk formulae are a primary bottleneck to seasonal prediction (Penny and Hamill, 2017). In other words, even if we had a perfect ocean model with perfect initial conditions, the information retained in the monthly ocean prediction would be degraded when propagated to the atmosphere due to uncertainty in estimating the true exchanges (Vecchi et al., 2014). Therefore, a priority in the coming decade must be to gather *in situ* estimates of air-sea-ice exchanges in the context of large-scale properties informing how to minimize errors in the bulk formulae parameterizations. Efforts such as YOPP, MOSAiC and ONR Marginal Ice Zone (MIZ) project are examples of projects aiming to address this gap.

We recommend further research to determine how best to represent these exchanges in a coupled forecasting system, with a focus on determining what aspects of boundary layer physics need to be resolved and what can be skillfully parameterized. For parameterized physics, we require that process studies be carried out to determine parameterizations and parameterization coefficients, including identifying the observations that should be sustained to validate estimated fluxes. Addressing these areas are primary goals to enable weekly-to-seasonal skillful predictions in the polar regions.

## FORECASTING SYSTEM EXPERIMENTS

Understanding how assimilating more observations will impact modeled analyses and short-term forecasts is of fundamental importance as new coupled models are being developed. For instance, preliminary results using a regional coupled model [CAFS from the NOAA Earth System Research Laboratory (ESRL)] demonstrated that current ocean reanalyses do not include realistic representation of subsurface water masses relative to observations taken in 2015 and 2016 (including Arctic Heat data). Forecast experiments using a high-resolution fully coupled regional model show that these water masses impact sea ice evolution on synoptic time scales through upper-ocean mixing and heat flux at the ice–ocean interface. It is expected these effects will increase as sea ice continues to decline and surface heat flux processes increase. In addition, assimilating real-time ocean observations in the initial forecast conditions allows for the identification of biases in the coupled system, which are difficult to isolate when the ocean is allowed to drift away from the observed state. Some studies (e.g., Inoue et al., 2015; Lien et al., 2016; Xie et al., 2017) have begun to show the important potential impact of assimilating enhanced observations on model-based analyses and short-term sea ice forecasts. Additional focused research in this area would allow us to explore coupled model data assimilation issues, better understand physical processes, and assess model performance in comparison to non-coupled (atmospheric) model frameworks. In the next sections, we review a few results from operational centers.

### Impacts of Arctic and Antarctic Observations in U.S. Navy Coupled Ice-Ocean Models

The ability to forecast sea ice conditions is of crucial importance for maritime operational planning (Greenert, 2014). The current U.S. Navy operational sea ice forecast system is the Global Ocean Forecast System (GOFS) version 3.1. GOFS utilizes the Los Alamos Community Ice CodE (CICE) version 4.0 (Hunke and Lipscomb, 2008) sea ice model, which is two-way coupled with the HYbrid Coordinate Model (HYCOM) (Metzger et al., 2014) ocean model. The grid resolution is 1/12°, with horizontal resolution approximately 3.5 km at the poles. Atmospheric forcing is provided by the NAVy Global Environment Model (NAVGEM; Hogan et al., 2014). The precursor to GOFS 3.1 was the Arctic Cap Nowcast/Forecast System (ACNFS; Posey et al., 2015). ACNFS is a coupled ice/ocean model similar to GOFS with two main differences: (1) ACNFS only covered areas north of 40°N, and (2) in ACNFS HYCOM has 32 ocean layers compared to 41 in GOFS. An important part of both GOFS and ACNFS is the assimilation of observational data, which is accomplished using the Navy Coupled Ocean Data Assimilation (NCODA) system (Cummings and Smedstad, 2014).

Assimilation of observational data is performed to reduce errors in model forecasts that can result from many factors including non-linear processes that are not deterministic responses to atmospheric forcing, poorly parameterized physical processes, limitations in numerical algorithms, and limitations in model resolution. Polar observational data assimilation is an essential part of GOFS forecasts. Sea ice concentration observations are currently assimilated from the Defense Meteorological Satellite Program (DMSP) SSMIS and AMSR2. These observations are used in conjunction with the Interactive Multi-sensor Snow and Ice Mapping System produced by the U.S. National Ice Center (NIC; Helfrich et al., 2007). A full description of the assimilation process in GOFS and ACNFS can be found in Hebert et al. (2015).

**FIGURE 9 ∣:**
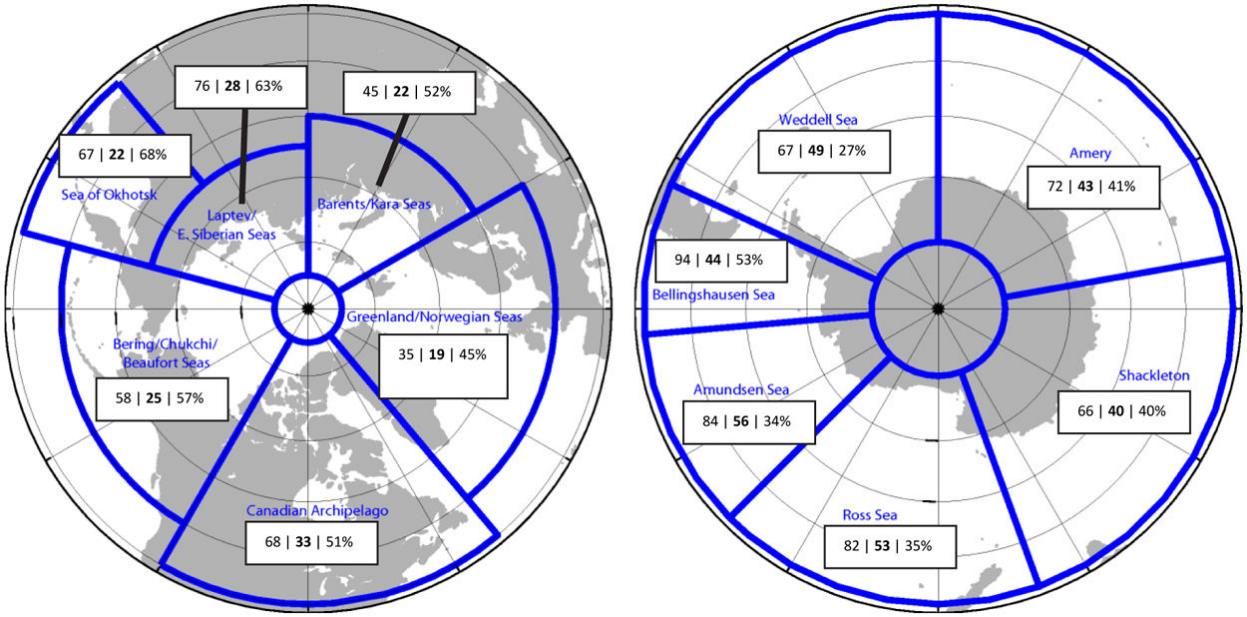
Ice edge error for individual regions (km). Each region contains three numbers. First number is ice edge error without assimilation. Second bold number is error with assimilation. Third number is percent improvement with assimilation. In the Arctic the overall reduction in ice edge error with observational data assimilation is 31 km (56%); in the Antarctic the overall reduction is 28 km (37%).

The impact of data assimilation in the U.S. Navy models is significant. One measure that the U.S. Navy uses to determine the accuracy of the modeled sea ice edge (defined in Hebert et al., 2015) is the distance between it and the sea ice edge determined daily by analysts at NIC. The sea ice edge error over six regions in each hemisphere is shown in Figure 9 as well as the entire Arctic/Antarctic. GOFS was run for 1 year (2014) without data assimilation and compared to the current GOFS system with ocean and sea ice data assimilation, including sea ice concentration. The impact of assimilating these observations was to reduce the sea ice edge error in the Arctic by 56% (31 km) and in the Antarctic by 37% (28 km), with a reduction in sea ice edge error for each region ranging between 27 and 68%.

As model resolution increases, so does the need for high-resolution observations. SSMIS and AMSR2 are relatively coarse resolution (25 and 10 km, respectively) compared that of GOFS. Higher resolution (less than 1 km) sea ice concentration observations are available from the Suomi NPP Visible/Infrared (VIIRS). Although VIIRS observations can be obstructed by clouds, including high resolution VIIRS ice concentration into our data assimilation further reduced GOFS ice edge error by 19% (5 km) in the Arctic and 11% (4 km) in the Antarctic. This result points to the need for higher resolution sea ice concentration observations to use in model applications.

In an earlier study using ACNFS to examine the impacts of sea ice concentration observations in ship routing and planning in boreal winter (January–March), assimilating satellite ice concentration observations reduced the projected track an ice breaker would take to a ship near the sea ice edge by an average of 150 km versus not assimilating sea ice concentration observations. This improved the time for planning operations by 12 h and reduced the distance a ship needs to prepare to encounter ice by 212 km.

SIT observations are also important. Currently, pan-Arctic SIT observations on a daily basis are not available. Only limited satellite tracks per day are available that are aggregated on a monthly basis. In a recent study by Allard et al. (2018), ACNFS was re-initialized on March 15th using the March 2014 monthly CryoSat-2 thickness observations and integrated for 18 months. It showed a reduction in SIT bias by 0.75–0.97 m compared to ACNFS SIT without CryoSat-2 initialization. The impact of this one-time re-initialization was significant and work is underway to assimilate daily satellite track SIT observations on a daily basis.

### Sensitivity of Sea Ice Forecasting Skill to Ocean Mixing Around Antarctica

The rapid evolution of the sea ice cover can have important impacts on coupled environmental predictions through a variety of processes (Smith et al., 2013). These include the formation of leads and coastal polynyas, as well as changes in the ice cover along the marginal ice zone (MIZ). In these regions, the rapid formation, melt and advection of the sea ice cover can modify atmosphere-ocean fluxes on relatively short timescales. Interestingly, small-scale ocean variability has a role to play here as the timing and intensity of changes will be sensitive to the surface ocean mixing layer depth, water mass properties and mesoscale ocean circulation (e.g., Zhang et al., 1999).

**FIGURE 10 ∣:**
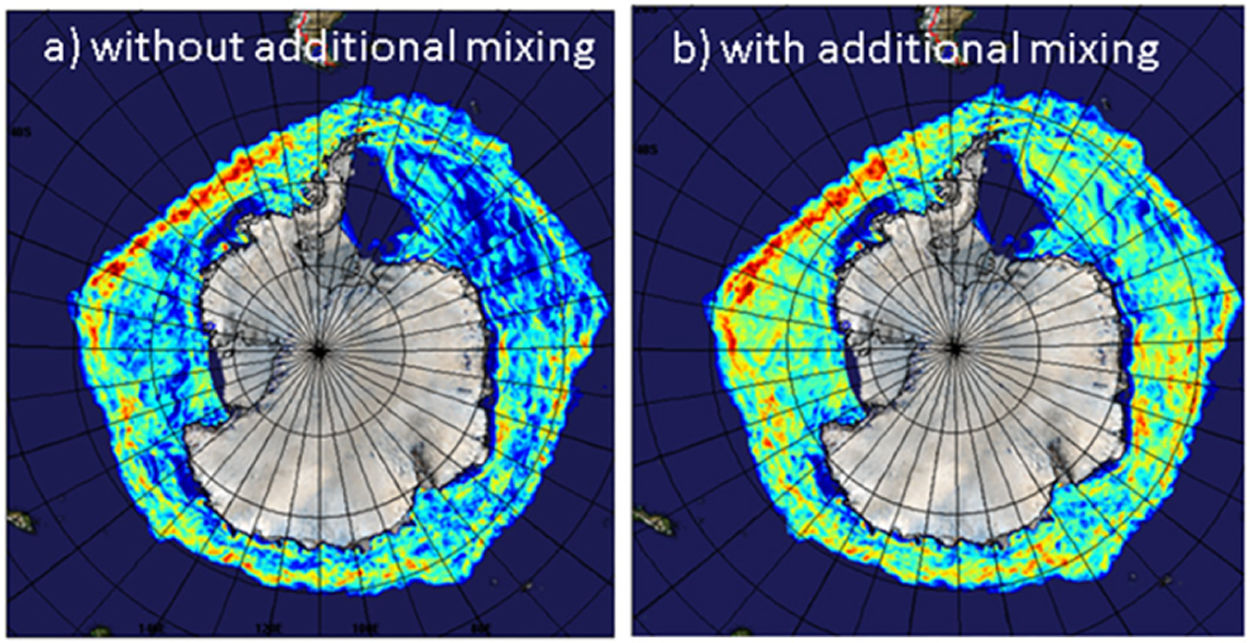
Sensitivity of sea ice forecasting skill to ocean mixing around Antarctica. Weekly 7 days sea ice forecasts from the Global Ice-Ocean Prediction System (GIOPS) running operationally at the Canadian Centre for Meteorological and Environmental Prediction are evaluated against analyses over the year 2011. The evaluation of forecast skill is restricted to points where the analysis has changed by more than 10% over the forecast period (7 days). Warmer colors indicate larger root-mean squared error (maximum of 0.3 for dark red) with zero error shown as dark blue. Panels **(a)** and **(b)** show the forecast skill for experiments without and with additional ocean mixing respectively. Adapted from Smith et al. (2013).

As an illustration of the sensitivity of sea ice evolution to ocean mixing, an evaluation of the skill of two sets of sea ice forecasting experiments is shown in Figure 10. The first set uses the standard configuration of the Global Ice-Ocean Prediction System (GIOPS; Smith et al., 2016) running operationally at the Canadian Centre for Meteorological and Environmental Prediction. GIOPS combines the Systeme Assimilation Mercator (SAM2) ocean analysis system with a 3DVar ice analysis (Buehner et al., 2013) to produce daily 10-day forecasts using the NEMO ocean model at 1/4° resolution coupled to the CICE ice model. The second set of experiments is identical to the first with the parameterization for surface wave breaking deactivated. Figure 10 shows the 7-day forecast skill evaluated against 3DVar ice analyses from weekly forecasts over 2011. The verification method used here (Lemieux et al., 2016) restricts the error evaluation to areas where the ice concentration analysis has changed by more than 10% over the forecast lead time (i.e., 7 days). This verification method has the advantage that it focuses the evaluation on ‘hot spots’ of activity, predominantly in the marginal ice zone.

From Figure 10 it can be seen that a small modification to the ocean vertical mixing can have a first order impact on the ice forecast errors. Interestingly, while the surface wave breaking parameterization degrades ice forecast skill, it does lead to an improvement in water mass properties over ice-free waters (as evaluated against Argo profiles; not shown). This is perhaps not surprising given that the mixing regime in polar regions is quite different from that at lower latitudes. This highlights the need for an expanded under-ice ocean monitoring program to be able to adequately model vertical mixing and constrain water mass properties and mixed layer depths.

### Impact of Temperature and Salinity Profiles in the CMEMS Arctic MFC

There have been special observing periods of the Arctic and Antarctic in the past, in particular the successive International Polar Years (IPYs), the latest of which took place in 2007–2009 (with a gradual ramping up of ocean observing systems in the preceding years). Looking back at the impact of a recent IPY in a period with similar low-ice-coverage conditions in the Arctic, expanded sea ice in the Antarctic, and similar satellite coverage as today can provide another measure of the expected impact of the YOPP Special Observing Periods.

In the Arctic, the TOPAZ4 reanalysis is based on a regional configuration of the HYCOM model coupled to an early version of the CICE model at 12 km resolution. It assimilates both satellite and *in situ* observations using an Ensemble Kalman Filter (Sakov et al., 2012). As of 2018, the TOPAZ4 reanalysis system is almost identical to the real-time physical forecasting system used in the Copernicus Arctic MFC and operated by MET Norway (Melsom et al., 2015; Xie et al., 2017). It assimilates the same types of ocean and sea ice observations: along-track sea-level anomalies from altimeters, sea surface temperatures, sea ice concentrations and drift and *in situ* temperature and salinity profiles.

**FIGURE 11 ∣:**
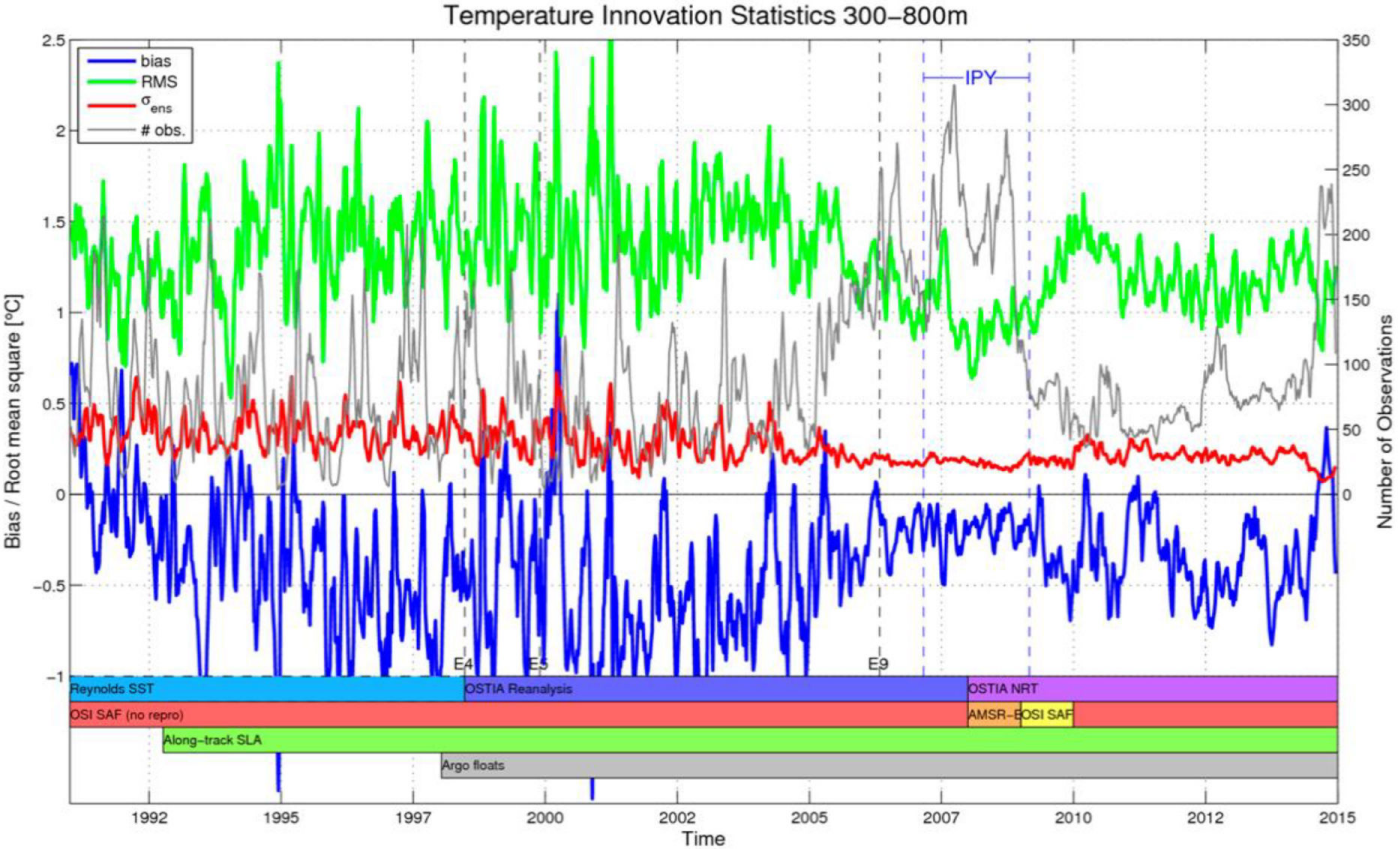
Time series of TOPAZ4 data assimilation diagnostics across the 24-year reanalysis for all temperature profiles in the depths 300–800 m in the whole Arctic. The blue line is the bias, the green line is the related standard deviation (Root Mean Square), the red line is the ensemble spread, and the gray line the number of temperature observations, increasing during the IPY. The other vertical lines and the bottom bars indicate changes of the other observation data sources and modifications of the data assimilation system.

Ocean models have well-known limitations in simulating the advection of Atlantic Waters into the Arctic (Ilicak et al., 2016), which for a model like TOPAZ4 results in a typical cold bias of the 300–800 m water temperature by 0.5 K (see Figure 11, blue line) and regional differences by 1.5 K typically (green line). During the IPY time period, the assimilation of ITPs is able to constrain this bias down to 0.1 K and reduce the regional differences below 1 K. The end of the IPY in 2009 relaxes the constraint and the bias and RMS errors return to larger values, although slightly lower than before the IPY (i.e., there is no “dynamical shock” after the IPY stops). Even though the quantitative impact on the TOPAZ4 system is dependent on the practical setup of the model and its assimilation scheme, the qualitative behavior may apply to other forecasting systems based on similar types of models and data assimilation schemes and indicates that a density of ITP profiles at least equal to that of the IPY should be sustained continuously to constrain efficiently the Atlantic Water properties in the Arctic.

### NOAA-ESRL/CIRES Coupled Arctic Forecast System

NOAA ESRL has provided experimental, daily, 10-day forecasts of Arctic weather and sea ice evolution to stakeholders during freeze-up seasons since 2015 and daily forecasts year-round starting on February 14, 2018. CAFS produces high-resolution (10 km) regional coupled-model Arctic forecasts using global 0.5° GFS forecast fields for lateral forcing. The current configuration of the model includes the POP2 dynamical ocean model (adapted from Maslowski et al., 2012), the CICE5 sea ice model (Hunke et al., 2015), the NCAR CLM4.5 land model, and the WRF3.6 ARW atmospheric model, coupled by the NCAR CPL7 coupler. The domain is the Arctic basin and surrounding coastal regions, including Bering Strait, to provide model guidance for the National Weather Service (NWS), and Fram Strait, to include the complete planned Multidisciplinary drifting Observatory for the Study of Arctic Climate campaign (MOSAiC) domain.

The CAFS forecasts are being used by NOAA ESRL to identify sources of skill on sub-seasonal time-scales due to coupled ocean-ice-atmosphere processes and by stakeholders as model guidance for sea ice forecasts. Real-time CAFS products are made available to the community^12^. Figures and animations from the 10-day forecasts are provided for sea ice, atmosphere, and ocean variability, as well as, an archive of model output for users to download. These forecasts are being used for model guidance by the NWS Alaska Sea Ice Program, the NOAA Arctic Testbed, the U.S. National Ice Center, and by the U.S. Navy and NOAA for operations during Arctic campaigns.

Coupled Arctic Forecast System forecasts use initial conditions that ingest SIT measurements from ESA’s CryoSat-2 and SMOS satellites and the NASA Jet Propulsion Laboratory Multi-Scale Ultra-high Resolution Sea Surface Temperature (MUR) SSTs and sea ice concentration. The MUR SSTs are used to initialize the ocean mixed layer with a mixed-layer depth diagnosed from the model. In order to identify whether using these satellite products in the initial conditions increases the skill of the 10-day forecasts, a series of 10-day hindcasts were performed for the time period of the ONR SeaState DRI, Oct. 1-Nov. 5 2015. The hindcasts are setup exactly like the real-time forecasts except the lateral boundary conditions are the GFS analyses instead of the GFS forecasts, in order to identify potential model biases. In addition, similar to the forecasts, hydrometeor mass and number are initialized with fields from the first day of the previous day’s hindcast to reduce spin-up time of cloud fields.

**FIGURE 12 ∣:**
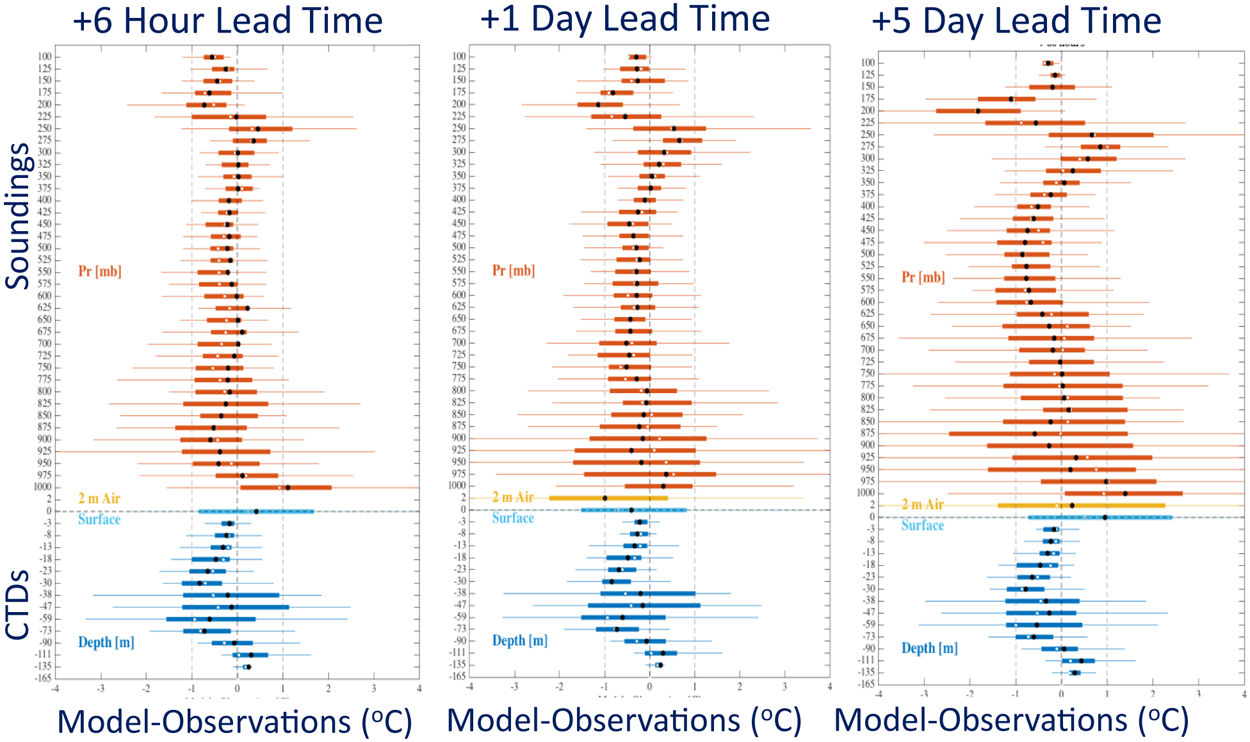
Box and whisker plots of CAFS forecast temperature errors in the full atmosphere-ocean column at 6 h, 1 day, and 5 days lead times compared to radiosondes (red) and CTDs (blue) from the R/V Sikuliaq during the ONR SeaState campaign October 1 – November 5 2015. Note, the vertical scale is model levels.

Intensive measurements were taken of the ocean, surface, and atmospheric state during the SeaState campaign. This provides for a comprehensive observational database for model validation. Figure 12 shows the model error of the ocean-atmosphere state at the location of the R/V Sikuliaq over the 6-week campaign at lead times of 6 h, 1 day, and 5 days. At a 6-h lead time, there are equivalent errors in the ocean and atmosphere, less than 0.5°C. Due to the initialization of the ocean mixed-layer with satellite SSTs, errors in the ocean grow slowly up to 5-day lead times. However, differently in the atmosphere, errors in the lowest 2 km grow rapidly with errors greater than +1°C in the near-surface temperatures and errors greater than −1° C at the top of the atmospheric boundary layer. This is an indication that the model is unable to maintain the observed boundary layer stratification and rapidly evolves into a less-stable state. It would not have been possible to identify this model bias without initialization of the ocean/ice state. Process studies are currently underway to identify if this bias is due to cloud processes or boundary layer parameterizations.

### Quantitative Network Design

The Quantitative Network Design (QND) approach constitutes a computationally efficient alternative to Observing System Experiments (Fujii et al., 2019). The approach can be used to inform the design of observing networks or space missions. QND evaluates a set of observations (network) in terms of its constraint on a target quantity, i.e., a quantity of interest. This evaluation is performed in a modeling system that is capable of simulating counterparts of the observations and of the target quantity from a set of unknowns in the system (control variables). For a detailed description of the formalism we refer to Kaminski and Rayner (2017). Briefly, it proceeds in two steps: In the first step, the observational information is used to infer the uncertainty in the posterior control vector, *C(x)*, that is consistent with the observational uncertainty, *C(d)*, and the prior uncertainty of the control vector, *C(x_0_)* [Equation (1)]. In the second step the uncertainty in the control vector is mapped onto an uncertainty in the target quantity, σ(y) [Equation (2)]. Both steps use appropriate sensitivities (linearizations/response functions) of the modeling system. The first step, an inversion step, is formalized by

(1) $$C(x{)}^{-1}=M^{\prime T}C(d{)}^{-1}M^{\prime }+C(x_{0}{)}^{-1},$$

and the second step by

(2) $$\sigma (y{)}^{2}=N^{\prime }C(x)N^{\prime T}+\sigma (y_{\mathrm{mod}}{)}^{2},$$

where ***M′**(**N′**)* denotes the sensitivity of the vector of observations (the target quantity) with respect to the control vector, as simulated by the modeling system, and σ*(y*_mocd_*)* is the residual uncertainty in the simulation of the target quantity that remains even for a perfect control vector. The approach represents a network through observational locations and times and the observational uncertainty, but does not require real observations. Consequently, it can evaluate hypothetical networks/space missions based on assumed instrumental specifications and space-time coverage. Observations can range from point-scale (*in situ*) to gridded data sets or level-1 satellite data, if appropriate forward models/observation operators mapping the model’s state variables onto the respective data stream (Kaminski and Mathieu, 2017) are available.

Historically, QND was first applied by Hardt and Scherbaum (1994) to optimize the locations of a set of seismic sensors. Rayner et al. (1996) applied the approach to the *in situ* sampling network for atmospheric CO_2_. For the physical sea ice-ocean system in the Arctic domain, Kaminski et al. (2015) applied the approach to evaluate idealized flight transects derived from NASA’s Operation IceBridge airborne altimeter ice surveys. Target quantities were 10-day to five-month forecasts of snow and ice volumes over areas relevant for maritime traffic (along the Alaskan coast) and offshore resource exploration (Chukchi Sea). The control vector was composed of physical constants in the model’s process representations as well as initial and boundary conditions.

In an activity funded through ESA’s Support to Science Element as a contribution to YOPP, Kaminski et al. (2017) constructed the Arctic Mission Benefit Analysis (ArcMBA) system. The system exploits the fact that model sensitivities at observational times and locations as well as the target quantities can be pre-computed, so that the actual assessment of a data set requires only matrix multiplications and inversions [Equation (1) and Equation (2)]. This means the assessment can be performed so fast that the ArcMBA system could be used as an interactive tool to assist decision makers, for example, in a meeting. Currently the system uses pre-computed model sensitivities ***M′*** for observations of SIT, sea ice freeboard, radar freeboard, laser freeboard, and snow depth along with precomputed model sensitivities ***N′*** for forecasts of snow and ice volumes for three regions along the Northern Sea Route (North-East Passage) respectively denoted by West Laptev Sea, Outer New Siberian Islands, and East Siberian Sea. These sensitivities were computed with the Max-Planck-Institute Ocean Model (MPIOM, Jungclaus et al., 2013) in a global configuration with high resolution over the Arctic (Niederdrenk, 2013). The control vector is composed of physical constants in the model’s process representations as well as initial- and boundary conditions.

Kaminski et al. (2017) applied the system to evaluate real and hypothetical remote sensing products. The real products were monthly SIT, sea ice freeboard, and radar freeboard, all derived from CryoSat-2 by AWI. These real products were complemented by two hypothetical monthly laser freeboard products (2 and 20 cm accuracy, respectively), as well as two hypothetical monthly snow depth products (2 and 15 cm accuracy, respectively). Target quantities are 4-week forecasts of snow and ice volumes over three target regions along the Northern Sea Route.

**FIGURE 13 ∣:**
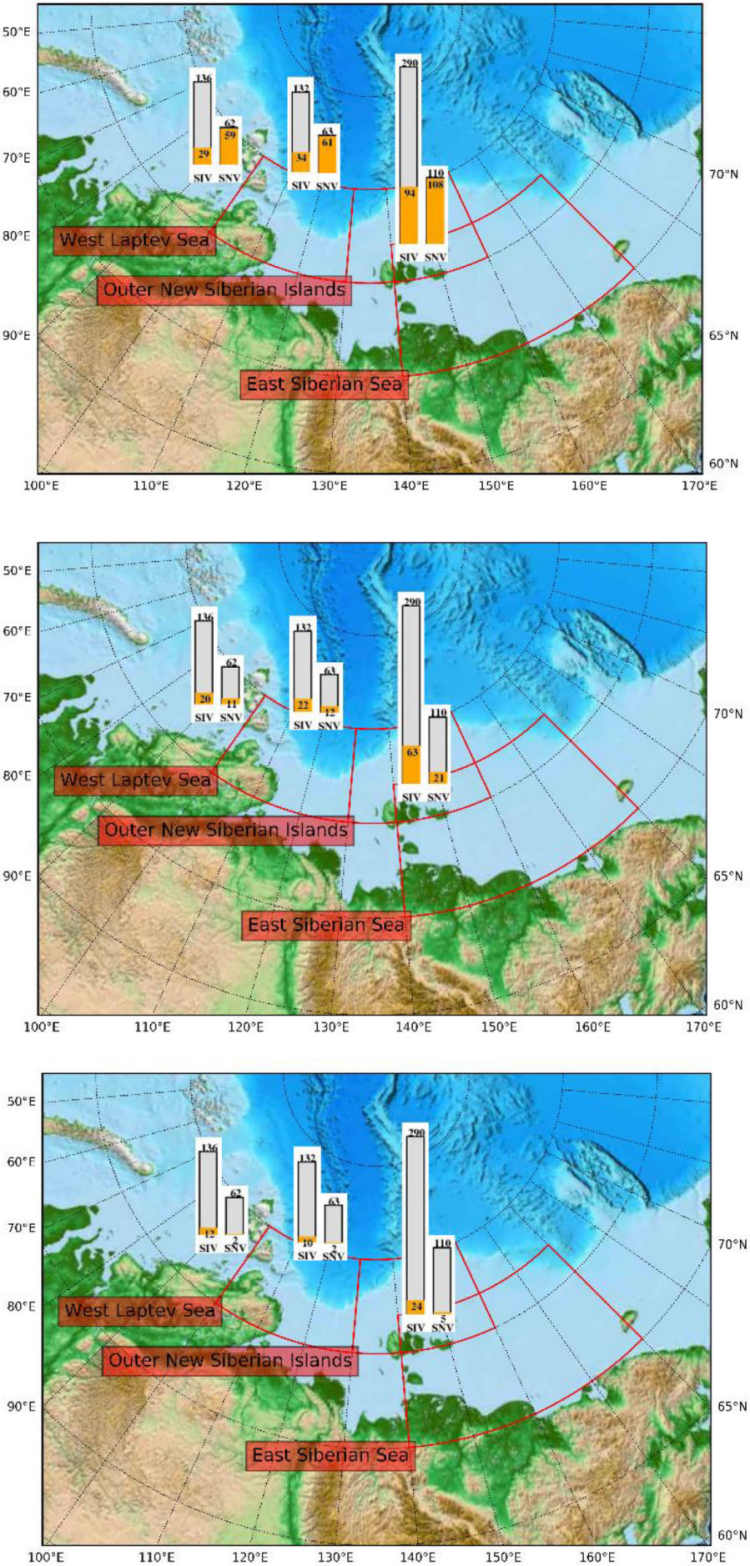
Evaluation of CryoSat-2 sea ice thickness product alone **(top)**, in combination with 15 cm accuracy snow depth product **(middle)**, and in combination with 2 cm accuracy snow depth product **(bottom)**. Prior (gray, no observations) and posterior (orange, sea ice thickness and snow depth products) uncertainties in sea ice volume (SIV) and snow volume (SNV) predictions for three regions along the Northeast Passage in km^3^.

As an example, Figure 13 shows the posterior uncertainty in snow and ice volumes over the three target regions, when the CryoSat-2 SIT product (Ricker et al., 2014) is used alone, in combination with the hypothetical snow depth product with 15 cm accuracy, or in combination with the hypothetical snow depth product with 2 cm accuracy. To sharpen the contrast between the observational scenarios, σ*(y*_mod_*)* (which acts as an offset) is set to zero (perfect model scenario). Comparison of the top and middle panels shows the added value of the hypothetical snow product, not only for the snow volume forecast but also for the sea ice volume (SIV) forecast. SIV is sensitive not only to initial SIT but also to initial ice concentration and snow depth, which are both constrained by the snow depth product. SIV is also sensitive to some of the process parameters that are constrained by snow depth, notably the ice strength, see Kaminski et al. (2017) for details. Comparison of the middle and top panels shows the added value of a higher accuracy in the snow depth product. Increasing the accuracy from 15 to 2 cm results in a reduction in uncertainty of the SIV forecast for the East Siberian Sea target region from to 63 to 24 km^3^.

The ArcMBA can be extended to cover further Earth Observation (EO) products and further target variables. In the setup used here, the model can simulate a range of sea ice-ocean variables in addition to those considered in the present study (e.g., ice drift, mixed layer depth, freshwater/sea surface salinity, SST, circulation). Switching to a more comprehensive model configuration would enable the investigation of yet further variables. For example the model can be operated with its biogeochemistry module HAMOCC activated (Ilyina et al., 2013) or in a mode coupled to an atmospheric general circulation model and thus enable the analysis of biogeochemical products/target quantities or Arctic mid-latitude linkages. The extension of ArcMBA by a terrestrial biosphere component is planned, which will allow joint assessment of ocean and land observations.

## INTERNATIONAL EFFORTS TO ADDRESS GAPS IN POLAR OCEAN OBSERVATIONS

Various international efforts contribute to coordinate and support the vast and complex polar observing networks. These networks are maintained by a collection of national and international efforts and scientific projects. These include national efforts outlined above, such as IAOOS, FRAMs and NorArgo as well as diverse multi-platform observing projects such as the US SODA (Stratified Ocean Dynamics of the Arctic) program (Lee et al., 2016) that include many of the types of platforms described in Section “*In situ* Observations of Temperature and Salinity” (WHOI ITPs, ALAMO floats, drifters, gliders). In the south, these efforts are coordinated through the Southern Ocean Observing System (SOOS; Newman et al., 2019) supported by the Scientific Committee on Ocean Research (SCOR) and the Scientific Committee for Antarctic Research (SCAR).

A clear need for a coordinated approach for the Arctic has arisen following the International Polar Year (IPY) and is being developed through a series of bi-annual Arctic Observing Summits (Murray et al., 2018). These summits contribute to a broad initiative by Sustaining Arctic Observing Networks (SAON) that has arisen from the International Arctic Science Committee. Recently, a framework for the development of an Arctic Region Global Ocean Observing System (ARGOOS) has been proposed by Lee et al. (2019). Additionally, the CLIVAR/CliC Northern Oceans Panel serves as an international forum for coordinating and strategizing activities on the role of the Arctic Ocean in the context of the global climate system from a coupled perspective and facilitates progress in developing new tools and methods to observe the Arctic Ocean and neighboring seas.

While these efforts aim to address a broad range of societal needs, in this section we describe several particular initiatives that work to address specific gaps in the observing system important for environmental prediction.

### INTAROS (INTegrated ARctic Observation System)

INTAROS is a research and innovation project under the European Union Horizon 2020 program, running from 2016 to 2021, with objective to build an efficient integrated Arctic Observation System in the Arctic. This requires collaborative efforts among many institutions to extend, improve and unify existing systems, which in many cases are designed and developed for specific scientific disciplines. INTAROS focuses on the *in situ* part of the observing systems, which represent the largest component of the integrated observing system. Satellite Earth Observation programs provide the most developed and operational components of the system, which are run by space agencies and satellite monitoring services such as Copernicus^13^. The satellite systems provide data for near real-time monitoring as well as for long-term climate observations. Validation of the satellite-derived variables is an important part of the operational services. *In situ* observations play an important role for this validation, but there is very limited access to such data from the Arctic Ocean. Furthermore, most of the ocean observations are only available in delayed mode, because they are provided by underwater moorings and seafloor observatories. Some platforms that operate at the surface can transmit data in near real-time and can therefore contribute to operational monitoring of sea ice and ocean variables.

INTAROS is multidisciplinary, implying that the observing systems encompass atmospheric, marine and terrestrial systems in the different regions of the Arctic. Marine observing systems are also divided into physical and biogeochemical components of the ocean surface (including sea ice), the water column and the seafloor. INTAROS contributes to all these components in collaboration with other observing programs and projects, by deploying new sensors and platforms to enhance the observing capacity in different Arctic regions. The collection of new *in situ* data under INTAROS started in 2017 and will continue through 2020, coinciding with and contribute to the extensive data collection in the MOSAiC program (see MOSAiC). Examples of INTAROS supported platforms include: IMBs deployed in the Central Arctic Ocean by the Finnish Meteorological Institute during Chinese Arctic expeditions (Lei et al., 2018); a network of Argo floats in the Nordic Seas in ice-free areas; bio-Argo floats in Baffin Bay together with Takuvik; and the Ferry-box transect between Norway and Svalbard^14^. INTAROS also supports data collection from the cargo vessel NORBJØRN that runs between Tromsø and Longyearbyen throughout the year. Data can be transmitted to land in near realtime and is available for operational monitoring. Ship of Opportunity is promising method to collect oceanographic data in Arctic waters, since there is a growing number of ships operating in the Arctic, especially tourist ships.

Arctic observing systems will benefit greatly from collaboration with local communities (Johnson et al., 2018). INTAROS is therefore working with Community-Based Monitoring (CBM) systems which are under development in many places in North America, Greenland and Russia. CBMs can be supplementary to scientific observations when indigenous and local people collect scientifically relevant data and made them available via websites^15^. Community-based monitoring can also provide valuable data that cannot be obtained from normal research and monitoring programs. In the circumpolar Arctic region there are a number of observing programs addressing sea ice, oceanographic data and observations of marine mammals and fish which are very important for the communities.

### EMODNET

Observations of the ocean are usually made for specific purposes. In order to save costs and improve marine knowledge, the European Union is now moving to a new paradigm where data are collected once and then used for many purposes. This paradigm is being implemented as part of the European Marine Observation and Data Network (EMODnet) consisting of a variety of organizations working together to assemble marine data and products, and to facilitate the dissemination of these resources to both public and private users. EMODnet is currently in its third development phase with the target to be fully deployed by 2020. Six Sea Basin Checkpoint programs were initiated as part of EMODnet, including the Sea Basin Checkpoint Arctic^16^, with the objective to examine the current status of Arctic data and to assess how fit-for-purpose the Arctic data are.

The project (2015–2018) aimed to identify problems and knowledge gaps and was organized in 10 challenges: wind farm sitting, marine protected areas, oil platform leak, climate change, coasts, fisheries management, fisheries impact, river input, bathymetry and alien species. Within the project, each dataset found (292 in total) was assessed on its spatial and temporal coverage, its accessibility and cost to access, the responsiveness and the temporal and vertical resolution. Also, each assessment report using a dataset (840 in total) was assessed on its adequacy for the project. For the oil spill challenge an oil accident was simulated. This demonstrated the necessity for rapid acquisition and inspection of ocean current and wind data in order to provide a reliable response capacity.

### MOSAiC

An important Arctic observing project called MOSAiC (Multidisciplinary Drifting Observatory for the Study of Arctic Climate; Shupe et al., 2018) is planned for 2019–2020. The German research icebreaker Polarstern will be frozen into the pack ice and over-winter in the Transpolar Drift to obtain measurements over a complete annual cycle. The MOSAiC sea ice platform provides an opportunity for greatly enhanced deployment of autonomous instrumentation and coordinated intensive field studies from research vessels, manned and unmanned aircraft, and distributed surface stations.

MOSAiC observations have been designed specifically to characterize the important coupled processes within the atmosphere-ice-ocean system that impact sea ice melting and freezing. These processes include heat, moisture, and momentum fluxes in the atmosphere and ocean, water vapor, clouds and aerosols, biogeochemical cycles in the ocean and ice, and many others.

The MOSAiC central observatory will be a manned, icebreaker ship-based ice camp with comprehensive instrumentation to measure coupled processes within the atmosphere, ice, and ocean. This intensive observatory will be embedded within a constellation of distributed measurements made by buoys, ice-tethered profilers, remote meteorology stations, underwater drifters, unmanned aerial systems, aircraft, additional ships, and satellites. These distributed observations will provide critical information on the spatial context and variability of key parameters, and allow for limited measurements in environments with sea ice of differing age, thickness, and concentration.

The additional observations from MOSAiC together with other sources during YOPP provide an ideal opportunity to assess the impact on forecast skill in environmental prediction systems through OSEs and process studies. Results will provide a quantitative baseline for use in decisions regarding how to configure a sustained Arctic observing system appropriate for the needs of environmental prediction.

### Year of Polar Prediction

The need for improved environmental predictions (i.e., atmosphere, ice and ocean) has motivated an international effort called the Polar Prediction Project (PPP^17^). The PPP was created under the auspices of the World Meteorological Organisation’s World Weather Research Programme (WWRP) to “promote cooperative international research enabling development of improved weather and environmental prediction services for the polar regions, on time scales from hourly to seasonal.” YOPP is a PPP flagship activity (Jung et al., 2016) with a core phase during 2017-2019, with the overarching goal to “Enable a significant improvement in environmental prediction capabilities for the polar regions and beyond, by coordinating a period of intensive observing, modeling, verification, user-engagement and education activities.” In particular, there are a series of Special Observing Periods for both the Arctic and Antarctic to improve the polar observing system to provide better coverage of high-quality observations in a cost effective manner, primarily by carrying out Observing System Experiments. The Southern Hemisphere SOP occurred from November 2018 to February 2019, and included deployment of extra radiosondes and drifting buoys, as well as the prolongation of the Ocean Observatories Initiative surface mooring at 55°S. The Arctic SOPs were conducted for winter (February–March, 2018) and summer (July–September, 2018). A more comprehensive list of research activities is given by PPP Steering Group (2013, 2014). Additional observations gathered through field programs will also be used to improve our understanding of key polar processes relevant for improving prediction skill. A third Arctic SOP is planned for February–March 2020 as part of YOPP to capitalize on the MOSAiC observational effort.

The additional observations from MOSAiC together with other sources during YOPP provide an ideal opportunity to assess the impact on forecast skill in environmental prediction systems through OSEs and process studies. Results will provide a quantitative baseline for use in decisions regarding how to configure a sustained Arctic observing system appropriate for the needs of Environmental Prediction.

## RECOMMENDATIONS

The scarcity of observations, the unique balance of physical processes, the key importance of sea ice, and the rapidly evolving climate of the Arctic, and the uncertainties in Antarctic sea ice trends and carbon uptake lead to a number of scientific challenges for observations in the context of a polar prediction system. Addressing these challenges motivates the following recommendations:

1. The presence of a seasonal ice cover limits the availability of real-time *in situ* data in polar regions to assist operational requirements. While several technologies have been developed (e.g., ITPs, gliders communicating via acoustic modems) a comprehensive real-time ocean observing network able to supplement Argo for polar regions has yet to be put in place, hindering progress toward coupled polar prediction. It is therefore recommended that a network of ice-borne measurement systems be deployed and supported operationally in ice-covered areas. These platforms represent well-proven technologies for year-round data collection and near-real time data transmission via satellites.
2. Antarctic measurements are also needed, in particular, to evaluate changes that could be harbingers of continental ice melt. Recent studies also highlight the importance of measuring and understanding the intra-hemispheric ocean interactions on numerical weather prediction to climate time-scales (Foppert et al., 2017). Ice-borne observing systems that have proven their utility in the Arctic should now be adapted and tested in the Antarctic marginal ice zone.
3. Conditions are changing rapidly with the loss of summer sea ice extent in the Arctic and changing ice-cover patterns in Antarctic marginal seas. Phenomena long considered negligible in the Arctic and Antarctic may be becoming important (e.g., ocean waves – Cavaleri et al., 2012; Massom et al., 2018). The observing system needs to be reevaluated with this in mind. The retreat of the Arctic ice cover increases the area where open-water (or seasonally ice free) observing systems (e.g., gliders, Argo) can operate providing the possibility in future to provide a substantial amount of near-real time data for polar prediction systems.
4. Ships of opportunity present a promising method to collect oceanographic data in polar waters, since there is a growing number of ships operating in these regions. Moreover, community-based monitoring can also provide valuable data that cannot be obtained from normal research and monitoring programs. These atypical observing methods should be encouraged and enhanced to provide a low-cost expansion to the *in situ* observing system.
5. *In situ* observations are routinely used for the calibration of remote sensing products over much of the globe, with fewer such calibrations made in polar regions due to lack of *in situ* observations. Studies have shown the large benefits that such calibrations can have (e.g., Castro et al., 2016; Kwok et al., 2017). The availability of near-real time *in situ* measurements could be used to improve the quality of satellite products and thus on downstream environment predictions that assimilate them. Additional efforts involving multi-platform calibration are needed to improve the quality of remote sensing products.
6. The increasing maturity of satellite sea-ice thickness winter-time products merging several sensors (e.g., CryoSat-2 and SMOS) and its positive impact in preliminary assimilation experiments call for symmetrical efforts in the Antarctic ocean, where such products do not exist at the moment.
7. Polar surface properties are often dominated by various forms of ice that vary rapidly on small spatial scales. Some remote sensing methods of ice properties (ice cover, ice thickness, snow depth on ice, albedo, crystal structure) are not mature and offer little and/or coarse-resolution information from within the ice, whereas *in situ* methods provide poor spatial coverage. Neither is currently able to address the need for high spatial and temporal resolution observations of sea ice deformation over large regions. Observations providing information regarding ice deformation and redistribution during ridging are also lacking. There is a need for high-resolution (km-scale) remotely sensed snow and ice property data for both the Arctic and Southern Ocean with sufficient temporal resolution to address these relevant features.
8. The value of polar observations for multi-range environmental prediction emerged during the last decade from a variety of impact studies. The importance of SIT initialization for seasonal forecasting, the significance of sub-surface initialization in ice-covered areas, are non-exhaustive examples that call for coordinated efforts (including QND, OSEs and Observing System Simulation Studies) to enhance the Arctic and Southern Ocean observing networks.
9. To date, few data withholding experiments (OSEs) or observation design experiments (OSSEs) have been undertaken for polar regions using real-time prediction systems. Performing such experiments using additional observations available during YOPP (or other periods of additional observational coverage such as IPY) is suggested to provide valuable information to guide the design of a sustainable real-time observing system for polar regions suitable for environmental prediction. In particular, multisystem exercises shall be encouraged to gain robustness in the observation impact assessments.
10. Polar environmental prediction using coupled atmosphere-ice-ocean models is strongly sensitive to errors in fluxes across the surface interface and thus requires collocated information about the state of the atmosphere, sea ice and ocean, to be used for improving interface fluxes (i.e., coupled model validation) and eventually data assimilation. Direct flux covariance measurements, in particular, would be immensely valuable in constraining bulk parameterizations used to represent fluxes in models.
11. Open access to data, especially real-time data, is a critical capability for improved sea-ice and weather forecasting and other environmental prediction needs. The optimal observing system will no doubt include a suite of different instrument types, since no single platform can be optimized for the full range of observing needs (i.e., sub-surface and lower atmosphere), and hence multi-platform coordination will be necessary, including coordination with local community-based observatories. This means that real-time dissemination of *in situ* observations in polar regions to global data assembly centers must be prioritized in order to make the observational efforts suitable for environmental prediction applications.
12. International collaboration will continue to be key for facilitating deployment of polar ocean instrument systems, including the fielding of drifting and anchored buoys, floats and gliders and free, rapid dissemination of the resulting data.

## OUTLOOK

The relative remoteness and harsh environmental conditions over polar regions will always hinder efforts to provide adequate observations for polar prediction. Over recent years, we have seen improvements in observing technology and capabilities that create new possibilities for how to construct and maintain the polar ocean observing system. The technologies make an adequate polar ocean observing system feasible, but the question remains, is it worth the cost? YOPP aims to help address this question by coordinating international observing activities and fostering efforts to assess the impact of additional observations on environmental prediction skill, including impacts on downstream users and products. Following the YOPP core period (2017–2019), there will be a consolidation phase to assess these impacts and develop recommendations toward sustained polar observation. This effort will culminate in a YOPP Final Summit planned for summer 2022, providing an ideal opportunity for funding and implementation agencies to benefit from this community effort.

## Figures and Tables

**FIGURE 7 ∣:**
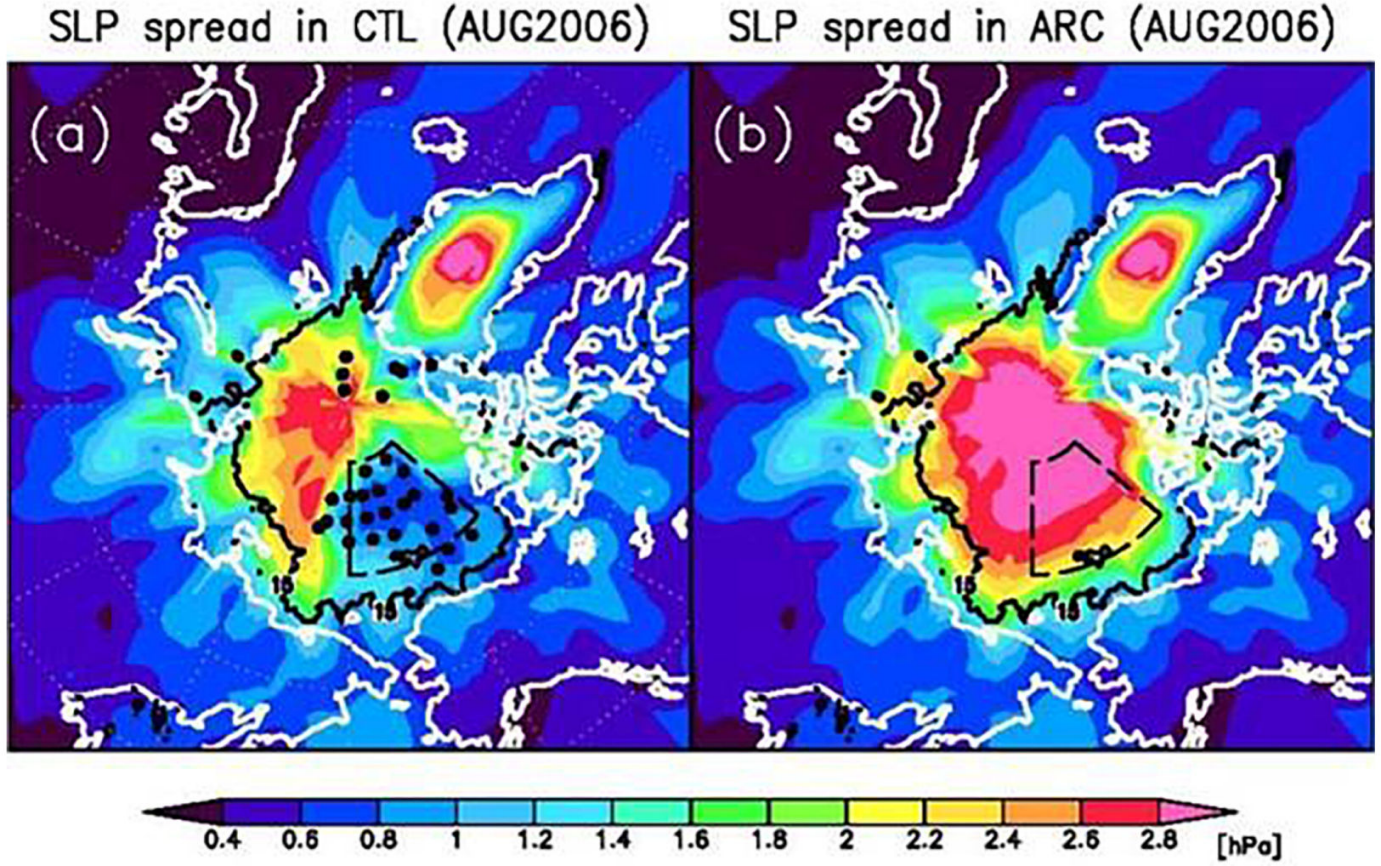
Standard deviation (SD) of sea level pressure measurements from various atmospheric reanalyses. The SD is low in areas where there are buoy observations **(A)**. The spread increases to cover the whole Arctic when the observations from the buoys are removed from the reanalyses **(B)** (Inoue et al., 2009).

## Footnotes

1 See http://iaoos.ipev.fr/ for more details.

2 See https://www.oc.nps.edu/~stanton/fluxbuoy/ for more details.

3 http://www.whoi.edu/itp

4 https://www.pmel.noaa.gov/itae/

5 https://www.pmel.noaa.gov/arctic-heat/data

6 http://www.greenedgeproject.info/

7 http://www.nke-instrumentation.com/

8 Naos, “Equipex Naos.” http://www.naos-equipex.fr/.

9 CORIOLIS, “Argo float data and metadata from Global Data Assembly Centre (Argo GDAC). Ifremer.” http://doi.org/10.17882/42182.

10 http://IABP.apl.uw.edu

11 http://www.ipab.aq

12 https://www.esrl.noaa.gov/psd/forecasts/seaice

13 https://www.copernicus.eu/en

14 https://www.niva.no/en/projectweb/norsoop

15 https://mkp28.wixsite.com/cbm-best-practice

16 http://www.emodnet-arctic.eu

17 http://www.polarprediction.net
